# Global, regional, and national neck pain burden in the general population, 1990–2019: An analysis of the global burden of disease study 2019

**DOI:** 10.3389/fneur.2022.955367

**Published:** 2022-09-01

**Authors:** Dong Woo Shin, Jae Il Shin, Ai Koyanagi, Louis Jacob, Lee Smith, Heajung Lee, Yoonkyung Chang, Tae-Jin Song

**Affiliations:** ^1^Department of Neurology, Mokdong Hospital Ewha Woman's University College of Medicine, Seoul, South Korea; ^2^Department of Pediatrics, Yonsei University College of Medicine, Seoul, South Korea; ^3^Parc Sanitari Sant Joan de Deu/CIBERSAM, Universitat de Barcelona, Fundacio Sant Joan de Deu, Barcelona, Spain; ^4^Institución Catalana de Investigación y Estudios Avanzados (ICREA), Barcelona, Spain; ^5^Faculty of Medicine, University of Versailles Saint-Quentin-en-Yvelines, Montigny-le-Bretonneux, France; ^6^Centre for Health, Performance, and Well-Being, Anglia Ruskin University, Cambridge, United Kingdom; ^7^Department of Neurology, Seoul Hospital Ewha Woman's University College of Medicine, Seoul, South Korea

**Keywords:** neck pain, global burden, prevalence, incidence, years lived with a disability, socio-demographic index

## Abstract

**Background:**

This study describes the global epidemiology and trends associated with neck pain. Global Burden of Disease data collected between 1990 and 2019 were used to determine the global burden of neck pain in the general populations of 204 countries.

**Methods:**

Global, regional, and national burdens of neck pain determined by prevalence, incidence, and years lived with a disability (YLD) from 1990 to 2019 were comprehensively analyzed according to age, gender, and socio-demographic index using the Global Burden of Disease Study 1990 and 2019 data provided by the Institute for Health Metrics and Evaluation.

**Results:**

Globally, in 2019, the age-standardized rates for prevalence, incidence, and YLD of neck pain per 100,000 population was 2,696.5 (95% uncertainty interval [UI], 2,177.0 to 3,375.2), 579.1 (95% UI, 457.9 to 729.6), and 267.4 (95% UI, 175.5 to 383.5) per 100,000 population, respectively. Overall, there was no significant difference in prevalence, incidence, or YLD of neck pain between 1990 and 2019. The highest age-standardized YLD of neck pain per 100,000 population in 2019 was observed in high-income North America (479.1, 95% UI 323.0 to 677.6), Southeast Asia (416.1, 95% UI 273.7 to 596.5), and East Asia (356.4, 95% UI 233.2 to 513.2). High-income North America (17.0, 95% UI 9.0 to 25.4%) had the largest increases in YLD of neck pain per 100,000 population from 1990 to 2019. At the national level, the highest age-standardized YLD of neck pain was found in the Philippines (530.1, 95% UI 350.6 to 764.8) and the highest change age-standardized YLD between 1990 and 2019 was found in the United States (18.4, 95% UI 9.9 to 27.6%). Overall, the global burden of neck pain increased with age until the age of 70–74 years, and was higher in women than men. In general, positive associations between socio-demographic index and burden of neck pain were found.

**Conclusions:**

Because neck pain is a major public health burden with a high prevalence, incidence, and YLD worldwide, it is important to update its epidemiological data and trends to cope with the future burden of neck pain.

## Introduction

Neck pain is a common pain that occurs in the human body and causes not only pain but also neck pain disability and economic problems (1). In 2016, among the 154 conditions, low back pain and neck pain had the highest health care expenditure in the United States with an estimated $134.5 billion (2). In addition, its cost included not only direct but also indirect ways such as loss of productivity. This constitutes a burden not only for the patient with neck pain but also for social and economic systems (3, 4). Despite the burden of neck pain, little is known about the burden of neck pain. In a 2017 global burden of disease (GBD) study, neck pain was present mainly in Nordic countries (5). Although some studies have investigated the burden of musculoskeletal symptoms at regional and global levels (6, 7), studies on neck pain are still lacking. In addition, epidemiological data on neck pain in each country has been investigated, but research on the global burden of neck pain reflecting recent trends is limited (8, 9).

With the increase in global aging, the prevalence and incidence of neck pain are also presumably increasing. Knowledge of the global burden of neck pain could aid in the prevention and/or management of patients at high risk of neck pain and inform policy decision making at the national level.

The present study aimed to investigate the global, regional, and national burden of neck pain including prevalence, incidence, and years lived with disability (YLD) between 1990 and 2019 according to age, gender, region, and country, based on analysis of GBD information provided by the Institute for Health Metrics and Evaluation (IHME). An additional aim was to investigate the burden of neck pain based on socio-demographic index (SDI), which reflects the development level of individual countries.

## Materials and methods

### Overview

The GBD Study is a systematic and comprehensive study of diseases worldwide. Based on data generated in this study, it is possible to compare and analyze the global, regional, and national burdens of diseases (10). GBD Study complies with the Guidelines for Accurate and Transparent Health Estimates Reporting (GATHER) statement (11). All information in the GBD Study is anonymized, therefore, no informed consent is required when analyzing data generated by this study. Our study was also approved by the Institutional Review Board at the Ewha Woman's University Seoul Hospital for data use (SEUMC202108006).

Detailed methods for obtaining information for non-fatal or fatal estimates have been described in a previous publication (12). Estimates for neck pain were acquired from surveillance systems of diseases, registries, survey microdata, health claims data, and systematic reviews of published and unpublished reports (12). Pubmed/Medline, CINAHL, Embase, WHOLIS, CAP abstracts, and SIGLE databases were investigated by IHME regardless of language, age, and gender to generate data for the GBD Study. Search terms “neck pain,” “cervical pain,” “neckache,” and “neck ache” were used to examine the database. Then, these terms were re-searched in combination with the following terms: “population sample,” “population study,” “population-based,” “cross-sectional,” “cross-sectional,” “prevalen^*^,” and “inciden^*^” (12). Not only systematic reviews from the above data sources but also data generated by the National Health Interview Survey and the National Health and Nutrition Examination Survey in the United States in addition to other nation-wide claim data were reviewed for the GBD Study (12, 13). Studies or datasets with a small sample size (<150), review articles, non–population sample studies, and studies in which subpopulations of the national population were not clearly defined were excluded by the IHME, which is in charge of the GBD Study (12, 13). These datasets were deposited in the Global Health Data Exchange, and the characteristics of the data were analyzed using DisMod-MR 2.1 to pool heterogenous data, a Bayesian meta-regression tool (14, 15). All rates are presented as age-standardized rates. Data are reported using 95% uncertainty intervals (UIs) and changes from 1990 to 2019 as percentages with 95% UIs.

After performing systematic and meta-regression analyses, the IHME uploads results to its website. We used data from the GBD's publicly provided website. All GBD research results can be freely accessed and downloaded from the GBD compare website and the Global Health Data Exchange website (GBD Compare; available at https://vizhub.healthdata.org/gbd-compare/ and Global Health Data Exchange, available at http://ghdx.healthdata.org/) (10). From this website, we selected the year 2019 and downloaded data and figures with neck pain (B.11.4) as the cause. GBD 2019 methods are described in detail on the GBD website and in a previous study (12). GBD 2019 is a multinational collaborative study of countries around the world. The GBD database is updated every year, and the most recent version provides the burden of diseases according to age, gender, and region (369 diseases and injuries in 204 countries and territories) from 1990 to 2019. Data acquisition and analysis in our study followed the methodology described on the GBD website. Our dataset for neck pain between 1990 and 2019 was obtained from the GBD website using GBD standards. Several articles have already been published in various disease fields using GBD data (5, 16–18).

### Case definition

In the GBD Study, neck pain is defined as pain occurring in the cervical spine and lasting for at least 24 h, regardless of accompanying referred pain in both arms. The anatomical location of neck pain is defined based on the definition recommended by the Bone and Joint Decade 2000–2010 Task Force on Neck Pain and its Associated Disorders (19). In the GBD Study, shoulder or neck pain is considered proxies for neck pain (12).

### Years lived with disability

The GBD of neck pain was evaluated as incidence, prevalence, death, disability-adjusted life years (DALYs), YLDs, and years of life lost due to premature mortality (YLLs) (20). DALYs were defined as the sum of YLDs and YLLs. YLD is defined as the individual sequelae prevalence of a disease multiplied by the disability weight, quantifying the severity of the sequelae as a number between 0 (indicating full health) and 1 (indicating death) (21). YLL is the number of deaths multiplied by the standard life expectancy at the time of death. Standard life expectancy is obtained as the lowest observed age-specific mortality rate among a world population of more than 5 million (20). There was no evidence of mortality related to neck pain disorder in the GBD data, and YLD and DALY were, therefore, the same. In this study, the term YLD is used instead of DALY. Disability weights were estimated from nine U.S. population surveys and an open Internet survey that asked respondents to choose the healthier option among random pairs of health conditions presented with brief descriptions of key characteristics (21).

### Socio-demographic index

To investigate the association between the level of development of regions or countries and neck pain disorder, the SDI was utilized (12). SDI is a composite indicator that measures the developmental level of each country. It is defined as 0 in the lowest case and 1 in the highest case, and it is calculated based on the lag-distributed income per capita, the total fertility rate for those younger than 25 years, and the average educational level of the population over the age of 15 years (18). In our study, the age-standardized prevalence and YLD for each region and YLD for each country were estimated according to the SDI.

## Results

### Global burden of neck pain

Prevalence, incidence, and YLDs of neck pain for both genders for 2019 are presented in Table 1 as counts and age-standardized rates. Globally, prevalent cases, incident cases, and YLDs of neck pain were 222.7 million (95% UI 179.2 to 281.0 million), 47.5 million (95% UI 37.5 to 59.9 million), and 22.1 million (95% UI 14.5 to 31.7 million), with an age-standardized point prevalence, incidence, and YLD of 2,696.5 (95% UI 2177.0 to 3375.2), 579.1 (95% UI 457.9 to 729.6), and 267.4 (95% UI 175.5 to 383.5) per 100,000 population, respectively. There was no significant difference in the prevalence (−0.48%, 95% UI −2.58% to 1.67%), incidence (-−0.46%, 95% UI −2.13% to 1.52%), or YLD (−0.34%, 95% UI −2.47% to 1.85%) between 1990 and 2019 (Table 1).

**Table 1:** Prevalence, incidence, and YLDs of neck pain in counts and age-standardize rate for both genders combined in 1990 and 2019, with percentage change between 1990 and 2019 by GBD region.

	**1990**		**2019**		
					**Percentage change in age-standardized rates between 1990 and 2019 (%)**
	**Counts (95% UI)**	**Age-standardized Rate (per 100k)**	**Counts (95% UI)**	**Age-standardized Rate (per 100k)**	
**Prevalence**					
Global	124472266.4 (98829382.62, 157046440.72)	2709.42 (2172.31, 3409.76)	222718452.87 (179236776.52, 281066237.18)	2696.52 (2177.01, 3375.19)	−0.48 (−2.58, 1.67)
East Asia	39077935.33 (30854063.71, 50082585.39)	3516.78 (2794.53, 4474.71)	69969608.64 (55367965.57, 89652879.08)	3554.35 (2856.64, 4488.84)	1.07 (−4.49, 6.63)
Southeast Asia	14451096.01 (11365729.56, 18534152.08)	4157.99 (3308.61, 5247.04)	29519839.49 (23009122.54, 38008673.53)	4179.34 (3322.07, 5283.27)	0.51 (0.24, 0.79)
Central Asia	779666.37 (617818.36, 1002005.7)	1419.08 (1131.09, 1792.57)	1280886.72 (1000274.41, 1651198.12)	1416.96 (1128.28, 1790.59)	−0.15 (−0.33, −0.01)
High-income Asia Pacific	3847414.56 (3018304.81, 4897806.17)	1909.07 (1513, 2415.25)	5310049.65 (4244869.36, 6635977.77)	1875.39 (1495.82, 2371.82)	−1.76 (−2.65, −0.93)
South Asia	12974352.47 (10144015.21, 16757403.52)	1625.99 (1303.63, 2055.41)	27311537.93 (21593375.13, 34993697.89)	1629.33 (1306.94, 2058.23)	0.21 (0.05, 0.38)
Central Europe	2063410.06 (1635332.78, 2610454.18)	1490.42 (1188.63, 1892.12)	2378063.49 (1905796.1, 2962295.84)	1496.91 (1195.48, 1903.14)	0.44 (0.2, 0.68)
Eastern Europe	4259319.84 (3372777.54, 5439620.57)	1643.66 (1320.14, 2078.64)	4603509.71 (3657500.82, 5817934.34)	1642.67 (1318.66, 2077.02)	−0.06 (−0.21, 0.07)
Western Europe	16337623.55 (13166545.69, 20406986.07)	3571.64 (2862.4, 4488.83)	20179970.47 (16278830.21, 25041519.12)	3543.64 (2837.92, 4454.27)	−0.78 (−1.91, 0.45)
Southern Latin America	914666.48 (726956.76, 1156760.2)	1934.45 (1539.71, 2448.45)	1463862.34 (1171284.17, 1833815.17)	1933.65 (1539.34, 2447.46)	−0.04 (−0.08, −0.01)
High-income North America	13261394.99 (10706548.2, 16616246.16)	4189.01 (3357.22, 5279.23)	22660148.6 (18732550.4, 27132560.04)	4900.74 (4067.39, 5915.68)	16.99 (9.18, 25.39)
Andean Latin America	369607.81 (291097.74, 478522.19)	1356.07 (1069.36, 1738.55)	832511.89 (654632.34, 1076336)	1356.71 (1069.51, 1739.72)	0.05 (0, 0.1)
Central Latin America	1713274.7 (1346534.36, 2221882.69)	1478.34 (1170.31, 1889.51)	3749817.03 (2943154.61, 4825059.02)	1478.61 (1169.3, 1891.75)	0.02 (−0.09, 0.13)
Tropical Latin America	2683565.42 (2102727.33, 3477135.44)	2236.01 (1766.06, 2866.81)	5591445.58 (4384612.74, 7211020.4)	2226.72 (1759.72, 2852.94)	−0.42 (−0.68, −0.18)
North Africa and Middle East	7095988.15 (5588332.9, 9132517.42)	3069.14 (2410.32, 3897)	17380168.05 (13535871.63, 22315482.61)	3066.65 (2407.82, 3894.35)	−0.08 (−0.32, 0.13)
Central Sub-Saharan Africa	390707.04 (303573.99, 506192.05)	1151.96 (909.24, 1472.08)	981974.76 (765564.85, 1276071.05)	1150.64 (907.7, 1469.02)	−0.11 (−0.29, 0.1)
Eastern Sub-Saharan Africa	1383661.82 (1080841.32, 1800958.85)	1228.04 (973.32, 1562.44)	3250444.71 (2539684.93, 4240126.9)	1231.37 (975.16, 1565.44)	0.27 (0.17, 0.41)
Southern Sub-Saharan Africa	490133.19 (383572.01, 633643.8)	1311.72 (1038.7, 1677.72)	935364.22 (732177.59, 1217556.08)	1316.1 (1042.06, 1683.3)	0.33 (0.16, 0.55)
Western Sub-Saharan Africa	1588223.18 (1252226.89, 2033872.51)	1298.49 (1031.73, 1657.89)	3907081.2 (3061236.48, 5079947.62)	1329.95 (1056.3, 1700.97)	2.42 (−0.04, 4.88)
Oceania	142154.99 (112128.83, 184830.68)	3207.76 (2555.48, 4078.55)	331821.66 (259593.59, 427598.49)	3209.74 (2556.51, 4080.99)	0.06 (−0.01, 0.14)
Australasia	245246.44 (194202.66, 308366.9)	1104.36 (871.2, 1399.33)	393873.45 (313157.13, 490273.09)	1059.12 (840.85, 1336.17)	−4.1 (−5.58, −2.77)
Caribbean	402823.98 (319004.04, 519403.75)	1356.7 (1069.82, 1739.21)	686473.29 (540972.67, 883813.99)	1357.33 (1070.6, 1740.2)	0.05 (0, 0.1)
**Incidence**					
Global	27650151.46 (21839122.68, 35190239.07)	581.74 (460.94, 737.68)	47528964.54 (37448843.73, 59936489.71)	579.09 (457.9, 729.64)	−0.46 (−2.13, 1.52)
East Asia	9196050.22 (7211128.6, 11762378.79)	796.03 (630.41, 1010.82)	15290226.66 (11998038.67, 19669322.36)	804.1 (638.38, 1020.47)	1.01 (−2.57, 4.78)
Southeast Asia	3324510.89 (2582246.85, 4283020.99)	892.82 (705.74, 1126.42)	6412627.16 (4999352.13, 8221511.06)	897.3 (709.29, 1133.99)	0.5 (0.26, 0.76)
Central Asia	186452.81 (145296.63, 237226.22)	326.81 (256.42, 413.18)	300561.98 (233910.28, 385242.37)	326.52 (256.1, 412.58)	−0.09 (−0.21, 0.01)
High-income Asia Pacific	843576.43 (663567.15, 1076220.87)	424.22 (334.34, 539.6)	1085782.41 (858963.95, 1353799.88)	413.79 (326.47, 525.14)	−2.46 (−3.09, −1.91)
South Asia	3223960.75 (2503146.51, 4151497.57)	384.41 (299.91, 483.81)	6597845.82 (5150566.8, 8422425.54)	384.98 (300.36, 484.57)	0.15 (0.05, 0.26)
Central Europe	473145.83 (373333.12, 599156.46)	346.63 (271.05, 438.38)	526037.45 (417177.86, 657314.95)	348.59 (272.57, 440.76)	0.56 (0.38, 0.74)
Eastern Europe	988834.8 (770350.73, 1249171.77)	387.54 (302.79, 487.94)	1044113.95 (818597.5, 1310466.47)	387.59 (302.54, 487.69)	0.01 (−0.1, 0.11)
Western Europe	2772554.49 (2217779.05, 3428428.81)	638.6 (504.76, 802.26)	3318754.52 (2625108.38, 4106299.86)	639.05 (505.78, 803.75)	0.07 (−0.89, 0.98)
Southern Latin America	189125.34 (148292.98, 239799.27)	396.88 (310.37, 501.51)	294219.85 (230877.84, 369571.33)	396.75 (310.3, 501.31)	−0.03 (−0.07, 0)
High-income North America	2531143.85 (2009720.12, 3199036.3)	811.2 (641.92, 1030.46)	3898342.46 (3147792.29, 4722527.88)	915.2 (736.57, 1122.21)	12.82 (7.11, 18.35)
Andean Latin America	90313.19 (70103, 116094.19)	313.34 (244.23, 397.23)	194773.58 (151509.11, 248119.56)	313.42 (244.25, 397.34)	0.02 (−0.02, 0.06)
Central Latin America	425654.08 (329981.26, 550211.99)	346.04 (271.49, 440.79)	881972.84 (687579.67, 1130334)	345.82 (271.1, 440.72)	−0.06 (−0.17, 0.03)
Tropical Latin America	635473.53 (492596.37, 823754.02)	503.05 (394.34, 641.9)	1250558.41 (977681.16, 1603774.7)	500.36 (392.45, 637.52)	−0.53 (−0.77, −0.31)
North Africa and Middle East	1620651.84 (1254946.48, 2092033.72)	650.81 (510.17, 833.75)	3842558.19 (2969837.34, 5062070.03)	649.2 (509.23, 829.21)	−0.25 (−0.52, −0.03)
Central Sub-Saharan Africa	97626.73 (74812.03, 124769.13)	268.66 (209.17, 341.02)	246043.83 (187967.55, 318436.78)	268.28 (209.08, 340.47)	−0.14 (−0.3, 0.03)
Eastern Sub-Saharan Africa	349917.84 (268550.93, 448754.61)	288.57 (226.23, 366.33)	822238.03 (630159.08, 1064031.63)	289.19 (226.79, 367.14)	0.21 (0.13, 0.33)
Southern Sub-Saharan Africa	122174.31 (95106.69, 157116.61)	309.28 (244.56, 391.73)	226778.7 (175057.02, 292595.77)	310.04 (244.92, 392.42)	0.25 (0.07, 0.48)
Western Sub-Saharan Africa	395147.77 (305023.59, 505704.14)	303.99 (239.51, 385.64)	977927.48 (747069.1, 1261457.29)	310.23 (242.34, 392.01)	2.05 (0.28, 3.84)
Oceania	32587.04 (25293.46, 42269.68)	675.1 (534.43, 853.56)	74588.48 (58068.25, 96779.09)	675.69 (534.88, 854.74)	0.09 (0.02, 0.15)
Australasia	55278.89 (43668.5, 69236.26)	251.27 (198.55, 314.81)	85899.58 (68491.04, 106698.4)	241.27 (192.37, 300.91)	−3.98 (−5.25, −2.84)
Caribbean	95970.82 (74751.55, 122090.18)	313.46 (244.31, 397.41)	157113.15 (122889.16, 198296.4)	313.51 (244.37, 397.34)	0.02 (−0.02, 0.06)
**YLDs**					
Global	12393477.83 (8128865.83, 17740323.75)	268.26 (176.71, 382.67)	22081323.13 (14508244.16, 31726932.92)	267.35 (175.53, 383.54)	−0.34 (−2.47, 1.85)
East Asia	3937613.77 (2558938.34, 5755034.55)	351.7 (229.25, 513.92)	6991872.96 (4541439.3, 10043872.72)	356.35 (233.22, 513.21)	1.32 (−4.26, 6.93)
Southeast Asia	1447224.48 (945236.25, 2088825.88)	412.28 (271.19, 590.34)	2951469.27 (1944453.78, 4240975.22)	416.13 (273.74, 596.53)	0.93 (0.3, 1.51)
Central Asia	78149.14 (50898.37, 114046.05)	141.4 (92.05, 203.66)	128309.54 (83542.74, 188387.45)	141.13 (91.9, 203.7)	−0.19 (−1.75, 1.4)
High-income Asia Pacific	383253.12 (251273.8, 556001.57)	190.24 (125.69, 275.57)	524073.31 (343739.73, 762845.02)	187.76 (124.65, 272.4)	−1.3 (−2.55, −0.07)
South Asia	1281202.65 (841619.93, 1875187.53)	158.49 (105.59, 228.7)	2695092.36 (1783438.42, 3919417.41)	159.65 (106.13, 231.23)	0.73 (0.03, 1.46)
Central Europe	203788.31 (134165.95, 294850.51)	147.51 (97.29, 212.2)	233685.18 (152893.22, 336638.74)	148.76 (98.07, 214.87)	0.84 (−0.21, 1.8)
Eastern Europe	418563.02 (276360.03, 607356.03)	161.97 (107.23, 234.61)	452371.21 (295617.42, 652101.5)	162.79 (107.9, 236.44)	0.5 (−0.51, 1.46)
Western Europe	1615146.09 (1087076.23, 2326364.25)	355.44 (237.29, 516.3)	1985044.77 (1322068.34, 2849095.51)	353.29 (234.75, 509.93)	−0.61 (−1.91, 0.8)
Southern Latin America	91051.26 (59742.9, 129900.97)	192.35 (125.93, 274.26)	144984.61 (95211.1, 207223.22)	192.09 (126.84, 274.22)	−0.14 (−2.21, 2.08)
High-income North America	1292392.48 (852563.8, 1838802.83)	409.68 (270.55, 586.24)	2188580.59 (1489072.06, 3091220.52)	479.11 (322.96, 677.58)	16.95 (8.95, 25.35)
Andean Latin America	37099.5 (23944.2, 53892.56)	135.05 (88.51, 194.96)	83265.63 (55078.22, 119749.24)	135.34 (89.56, 194.56)	0.21 (−2.14, 2.84)
Central Latin America	171454.54 (112419.89, 248720.03)	146.35 (97.26, 212.15)	372549.5 (246830.73, 539733.62)	146.6 (97.34, 211.78)	0.17 (−0.76, 1.13)
Tropical Latin America	266168.52 (173703.11, 388103.15)	219.98 (144.3, 319.69)	553219.44 (363652.26, 807726.87)	220.23 (144.35, 320.39)	0.11 (−0.92, 1.14)
North Africa and Middle East	707857.04 (466301.58, 1024803.57)	303.51 (201.51, 440.4)	1729128.39 (1136538.24, 2521211.89)	302.98 (201.54, 438.79)	−0.17 (-0.92, 0.51)
Central Sub-Saharan Africa	38698.54 (25275.12, 56027.24)	112.77 (74.41, 162.51)	98039.68 (63563.99, 142016.11)	113.43 (74.84, 163.66)	0.58 (−1.94, 3.37)
Eastern Sub-Saharan Africa	137895.25 (89830.51, 199729.7)	120.9 (79.65, 173.94)	325921.65 (210976.61, 472910.32)	121.86 (79.72, 174.64)	0.79 (−0.26, 1.77)
Southern Sub-Saharan Africa	48804.17 (31601.09, 70705.14)	129.3 (84.38, 184.1)	92207.86 (60668.53, 133909.99)	128.81 (84.61, 183.47)	−0.37 (−1.79, 1.06)
Western Sub-Saharan Africa	158528.33 (103543.01, 230294.55)	128.31 (85.2, 185.62)	391905.42 (252048.34, 564171.85)	131.86 (86.83, 189.14)	2.76 (0.18, 5.45)
Oceania	14203.26 (9118.59, 20348.02)	316.49 (206.51, 455.37)	33057.74 (21424.45, 47333.53)	315.99 (206.04, 451.38)	−0.16 (−1.79, 1.62)
Australasia	24165.97 (15866.96, 34772.52)	109 (71.33, 155.78)	38499.95 (25054.75, 55361.54)	104.56 (68.63, 149.93)	−4.07 (−7.58, −0.16)
Caribbean	40218.41 (26782.6, 57996.06)	134.89 (89.66, 194.27)	68044.07 (45122.31, 98537.75)	134.67 (89.48, 193.76)	−0.16 (−1.89, 1.53)

Data in parentheses are 95% uncertainty intervals (UI). GBD, global burden of disease, YLD, years lived with a disability.

### Regional burden of neck pain

High-income North America (4,900.7, 95% UI 4067.4 to 5915.7; 915.2, 95% UI 736.6 to 1,122.2; 479.1, 95% UI 323.0 to 677.6), Southeast Asia (4179.3, 95% UI 3322.1 to 5,283.3; 897.3, 95% UI 709.3 to 1,134.0; 416.1, 95% UI 273.7 to 596.5), and East Asia (3,554.4, 95% UI 2,856.6 to 4,488.8; 804.1, 95% UI 638.4 to 1,020.5; 356.4, 95% UI 233.2 to 513.2) had the highest age-standardized point prevalence, annual incidence, and YLD of neck pain per 100,000 population in 2019, respectively. Conversely, Australasia (579.1, 95% UI 457.9 to 729.6; 241.3, 95% UI 192.4 to 300.9; 104.6, 95% UI 68.6 to 149.9), Central Sub-Saharan Africa (1150.6, 95% UI 907.7 to 1469.0; 268.3, 95% UI 209.1 to 367.1; 113.4, 95% UI 74.8 to 163.7), and Eastern Sub-Saharan Africa (1,231.4, 95% UI 975.2 to 1,565.4; 289.2, 95% UI 226.8 to 367.1; 121.9, 95% UI 79.7 to 174.6) had the lowest age-standardized point prevalence, annual incidence, and YLD of neck pain per 100,000 population in 2019, respectively (Table 1). Age-standardized point prevalence, annual incidence, and YLD of neck pain per 100,000 population from 1990 to 2019 increased the most in high-income North America (17.0, 95% UI 9.2 to 25.4%; 12.8, 95% UI 7.1 to 18.4%; 17.0, 95% UI 9.0 to 25.4%, respectively), whereas these parameters decreased the most in Australasia (−4.1, 95% UI −5.6 to −2.8%; −4.0, 95% UI −5.3 to −2.8%; −4.1, 95% UI −7.6 to −0.2%, respectively) (Table 1).

### National burden of neck pain

Age-standardized point prevalence of neck pain per 100,000 population in 2019 was highest in the Philippines (5,333.5, 95% UI 4,269.7 to 6,740.7), United States (5,123.3, 95% UI 4,268.4 to 6,170.4), the United Kingdom (4,501.3, 95% UI 3,591.7 to 5,675.2), and lowest in New Zealand (871.0, 95% UI 702.2 to 1,082.5), Australia (1,092.7, 95% UI 861.2 to 1386.0), and Djibouti (1,138.5, 95% UI 898.1 to 1,454.09) (Supplementary Table S1).

Countries with the highest age-standardized incidence of neck pain per 100,000 population in 2019 were the Philippines (1156.3, 95% UI 917.1 to 1,461.5), Indonesia (965.0, 95% UI 763.7 to 1,218.9), and the United States (957.7, 95% UI 771.7 to 1,170.8), whereas New Zealand (212.7, 95% UI 170.6 to 263.0), Australia (246.2, 95% UI 194.9 to 307.7), and Djibouti (266.4, 95% UI 207.6 to 338.3) had the lowest rates (Supplementary Table S1).

**Figure 1:**
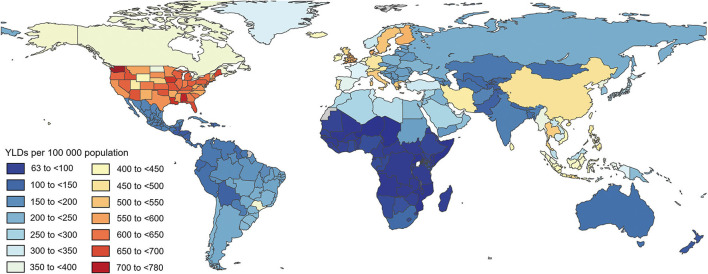
Age-standardized point YLD of neck pain per 100,000 population in 2019 by country. Generated from data available at http://ghdx.healthdata.org/gbd-results-tool. DALY, disability-adjusted life years.

Philippines (530.1, 95% UI 350.6 to 764.8), the United States (500.3, 95% UI 338.9 to 704.9), and the United Kingdom (446.9, 95% UI 302.0 to 636.9) had the highest age-standardized YLD of neck pain per 100,000 population in 2019, whereas New Zealand (86.1, 95% UI 56.5 to 122.9), Australia (107.9, 95% UI 70.8 to 155.1), and South Sudan (112.1, 95% UI 73.9 to 160.6) had the lowest rates (Figure 1 and Supplementary Table S1).

Percentage change in age-standardized point prevalence (ASPP), annual incidence (AI), and YLD of neck pain per 100,000 population from 1990 to 2019 differed between countries. The largest increases were in the United States (ASPP: 18.5, 95% UI 10.0 to 27.6%; AI 13.8, 95% UI 7.7 to 19.7%; YLD 18.4, 95% UI 9.9 to 27.6%), Malaysia (ASPP 11.8, 95% UI 2.7 to 23.1%; AI 9.8, 95% UI 2.9 to 18.0%; YLD 12.2, 95% UI 3.0 to 23.3%), and Nigeria (ASPP 5.5, 95% UI 0.9 to 10.3%; AI 4.8, 95% UI 1.4 to 8.2%; YLD 5.7, 95% UI 1.1 to 10.6%), respectively. In contrast, New Zealand (ASPP −25.2, 95% UI −32.5 to −17.9%; AI −23.3%, 95% UI −29.2 to −17.2%; YLD −24.9%, 95% UI −32.4 to −16.8%), Norway (ASPP: −17.9, 95% UI −23.7 to −11.2%; AI −12.5, 95% UI −15.8 to −8.3%; YLD −17.5, 95% UI −23.2 to −11.0%), and Taiwan (ASPP: Province of China, −7.8%, 95% UI −20.2% to 7.0%; AI −8.0, 95% UI −18.5 to 3.9%; YLD −7.8, 95% UI −20.5 to 7.3%) showed the largest decreases (Supplementary Table S1).

### Age-standardized YLDs

**Figure 2:**
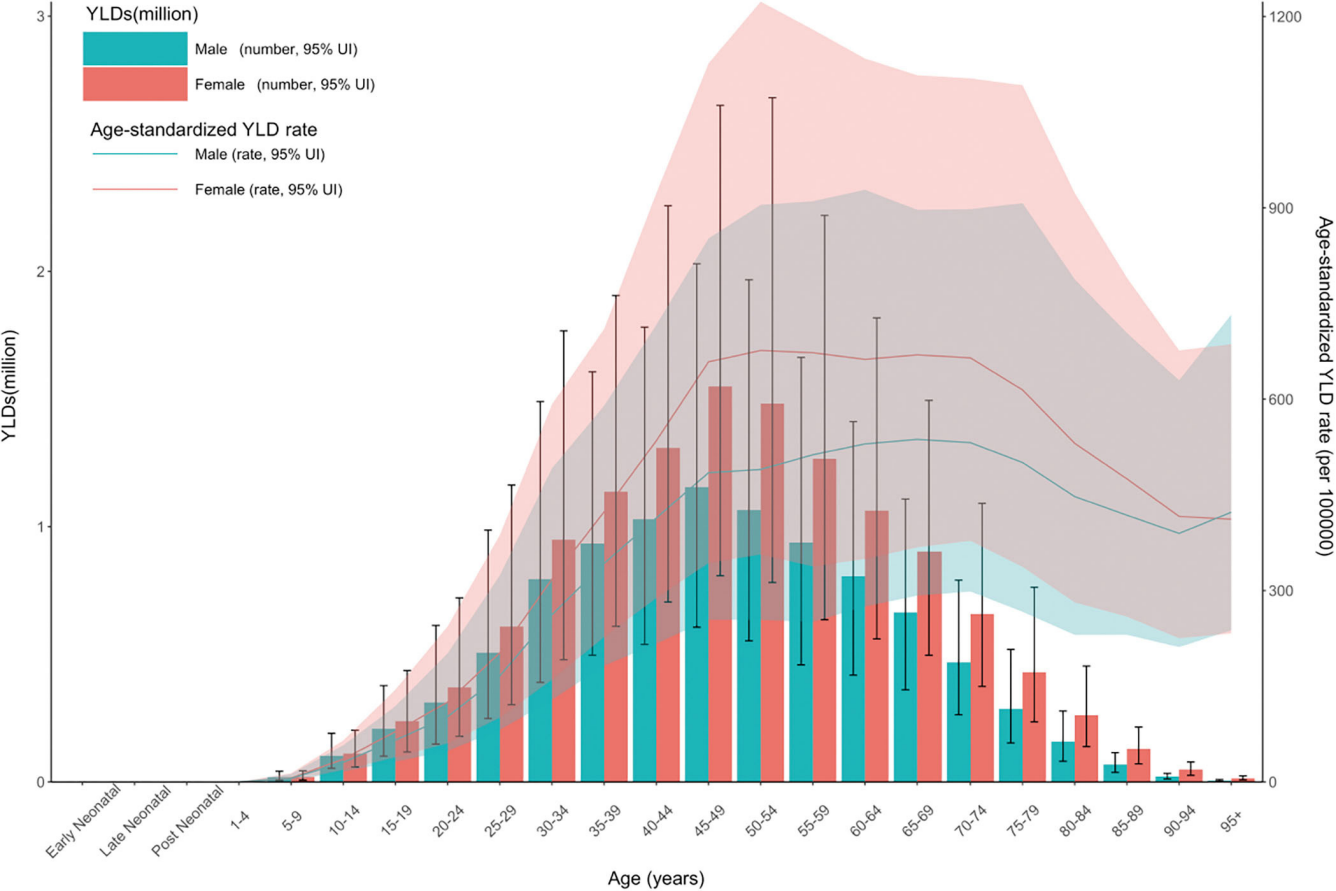
Age patterns by gender in 2019 for the total YLD number and age-standardized YLD rate of neck pain at the global level. Dashed lines (total YLD number) and transparent areas (age-standardized YLD) indicate 95% upper and lower uncertainty intervals, respectively. Generated from data available at http://ghdx.healthdata.org/gbd-results-tool. YLD, years lived with a disability.

Age-standardized YLDs (number and rate) for neck pain according to age and gender are presented in Figure 2. Age-standardized number of YLDs for neck pain was highest in both men and women aged 45–49 to 50–54 years. Considering the age-standardized rate of YLDs for neck pain, that of YLDs was the highest at 45–54 years of age. Overall, the global burden of neck pain was higher in women than in men, and neck pain increased in both men and women until the age of 70–74 years.

### Prevalence and YLD of neck pain according to SDI

**Figure 3:**
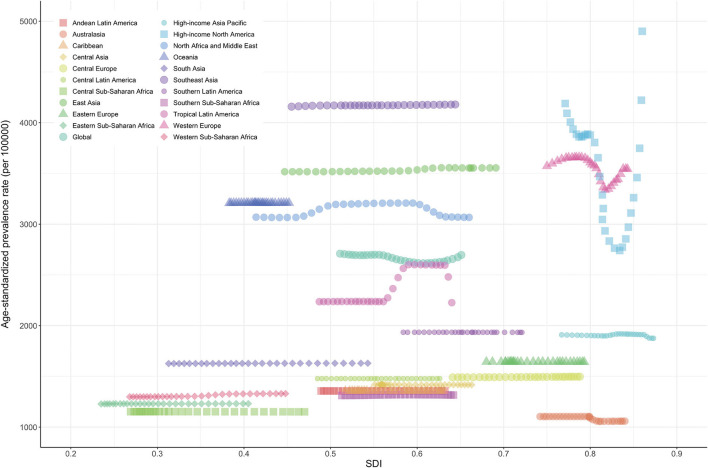
Age-standardized prevalence rate of neck pain for 21 regions according to SDI in 2019. Thirty points were plotted for each region and observed age-standardized prevalence for that region is shown. Generated from data available at http://ghdx.healthdata.org/gbd-results-tool. SDI, socio-demographic index.

**Figure 4:**
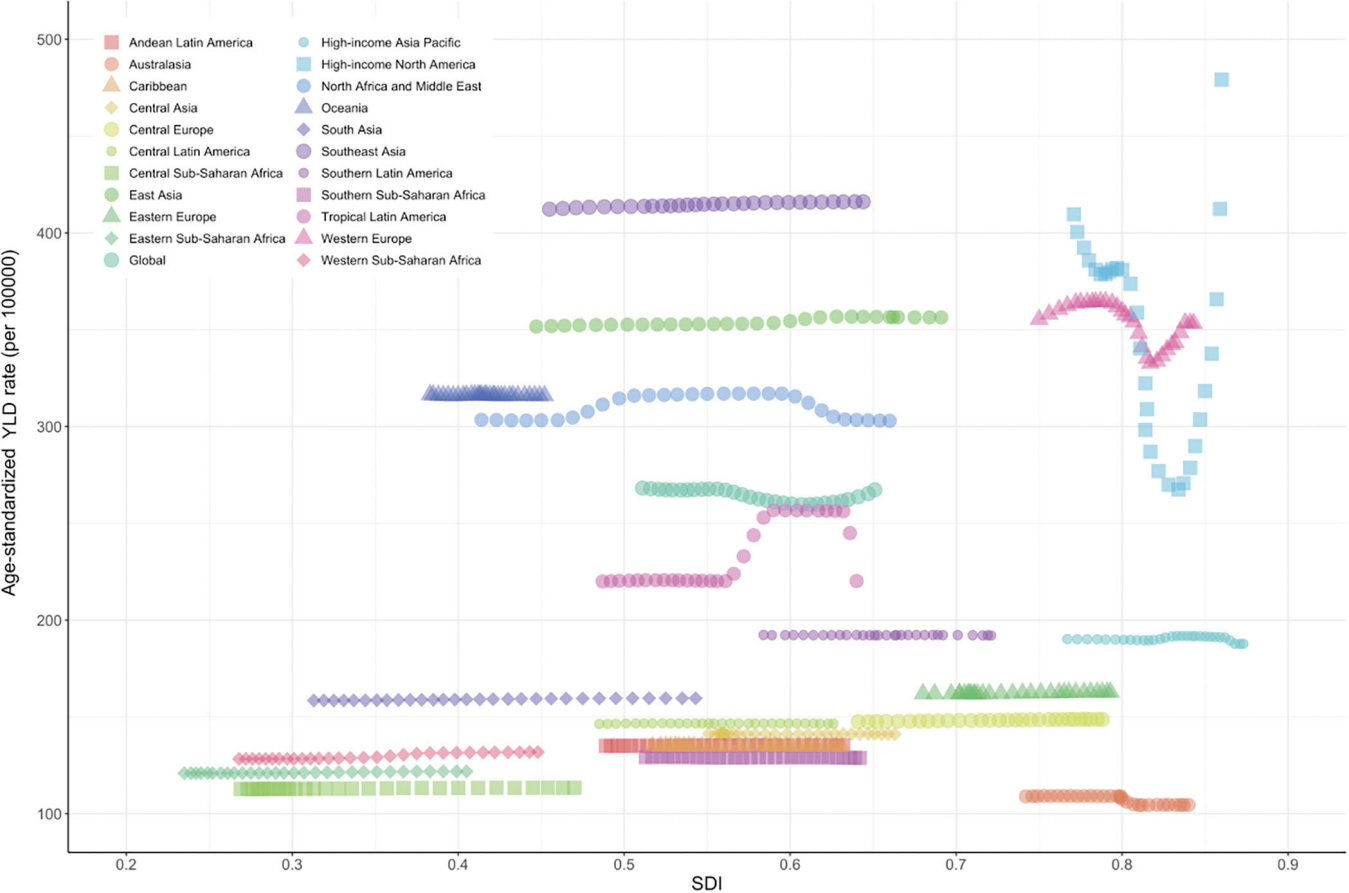
Age-standardized YLD rate of neck pain for 21 regions according to SDI in 2019. Thirty points were plotted for each region and observed age-standardized prevalence for each region is shown. Generated from data available at http://ghdx.healthdata.org/gbd-results-tool. YLD, years lived with a disability; SDI, socio-demographic index.

**Figure 5:**
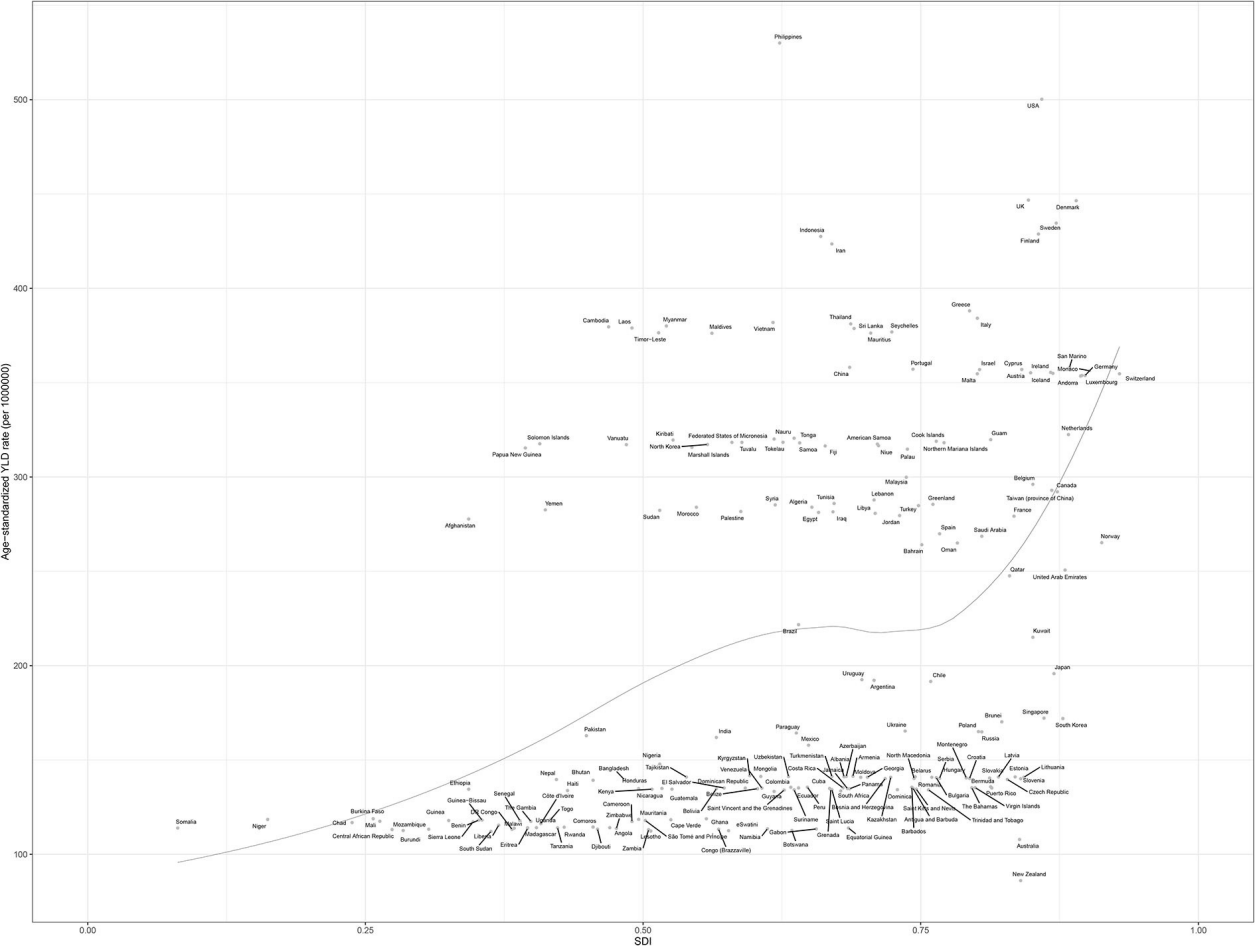
Age-standardized YLD rate of neck pain for 204 countries in 2019 according to SDI. Each point shows the observed age-standardized YLD rate for a specified country or territory in 2019. The black line represents expected values based on the sociodemographic index and disease rates in all locations. Generated from data available at http://ghdx.healthdata.org/gbd-results-tool. YLD, years lived with a disability; SDI, socio-demographic index.

In a previous study (5), the relationship between the burden of neck pain and SDI showed a positive trend. Also in our study, the burden of neck pain increased with higher SDI in general (Figure 5). Age-standardized prevalence rate (per 10,000) was relatively higher in high-income North America, Western Europe, and high-income Asia Pacific regions and lower in sub-Saharan Africa (Figure 3). Age-standardized YLD rate (per 10,000) for neck pain was also relatively high in high-income North America, Western Europe, and high-income Asia Pacific regions. By contrast, age-standardized YLD rate for sub-Saharan Africa was the lowest globally (Figure 4). Age-standardized YLD rate for each country according to SDI is presented in Figure 5. Age-standardized YLD rate was relatively high in Ireland, Australia, New Zealand, Finland, United Kingdom, Netherlands, United States, and Canada, all of which have a high SDI (Figure 5).

## Discussion

We reported the prevalence, incidence, and YLD of neck pain at the global, regional, and national levels from 1990 to 2019 and presented the age-standardized number and rate of YLD in 2019. The prevalence, incidence, and YLD of neck pain in 2019 were not significantly different from those in 1990. The global age-standardized prevalence of neck pain was 4.9% (95% CI 4.6 to 5.3) according to analysis of 2010 GBD data, 3,551.1 per 100,000 (95% UI 3139.5 to 3977.9) according to analysis of 2017 GBD data (5), and 2,696.5 per 100,000 (95% UI 2,177.0 to 3,375.2) according to analysis of 2019 GBD data.

Previous GBD studies (5, 22) have confirmed that neck pain is a common pain and causes disability and economic problems, and we have added a recent epidemiological trend to this. In the 2010 GBD analysis of neck pain (22), high-income North America showed the highest age-standardized prevalence, and East Asia had the highest age-standardized YLD at the regional level. In the 2017 GBD analysis of neck pain (5), the highest number and the highest increase in age-standardized incidence, prevalence, and YLD were in Western Europe (2017 Western Europe prevalence: 4636.1, 95% UIs 4077.2 to 5250.5; 2019 Western Europe prevalence: 3543.64, 95% UIs 2837.92 to 4454.27), while in 2019, it was in high-income North America (2017 High-income North America prevalence: 4900.74, 95% UIs 4067.39 to 5915.68; 2019 prevalence 4900.74, 95% UIs 4067.39 to 5915.68). At the national level, the Philippines had the highest age-standardized incidence, prevalence, and YLD for neck pain in 2019, but the highest increase in these variables was observed in the United States. In 2017 (5), Norway had the highest age-standardized incidence, prevalence, and YLD for neck pain while the United Kingdom had the highest increase rate in those areas. In year 2019 results of neck pain burden, United States of America, Malaysia, and Nigeria showed the largest increases, while New Zealand, Norway, and Taiwan presented the largest decreases. Based on the reliability of this GBD, collected and measured for 30 years, the numbers presented are meaningful. In previous studies (23–25), psychological factors (stress, anxiety, cognitive variables, sleep problems, social support, personality, and behavior) and biological factors (neuromusculoskeletal disorders, autoimmune diseases, genetic, gender, and age) are known as risk factors for neck pain, and therefore, future research goals should focus on the differences in policy approaches about these risk factor in these countries. In particular, there is a need for an analytical approach to various aspects of the low and constant neck pain burden in Australasia and Sub-Saharan Africa.

Overall, the YLD of neck pain is higher in women than men considering overall age. In previous studies, some studies considered female gender to be a risk factor for neck pain (26, 27) but others did not (24, 28, 29). Meta-analyses are required to elucidate if there is a gender disparity in neck pain. Women tend to have a higher prevalence of musculoskeletal disorders such as low back pain (30) and osteoarthritis (17), which have been attributed to female sex hormones, psychological factors, and sociocultural factors (31–34). YLD for neck pain was high in middle age, consistent with the results of previous studies (5, 22, 35). The high burden of neck pain in middle age is probably because this age group is active and takes on many tasks professionally. Furthermore, previous studies hypothesized that this relationship is due to the development of intervertebral disc degeneration and cervical spondylosis with age (36–39). The high YLD in men and women of middle age is not only a global medical problem, but also suggests the need for social and economic policies to reduce neck pain.

In our study, at the regional and national levels, age-standardized YLD of neck pain had a positive relationship with SDI. Although it is not possible to directly compare numbers due to different databases, definition methods, and analysis methods, a consistent finding is a higher burden of neck pain in countries with a high SDI (5, 9, 22, 40, 41). These results imply that the development level of regions or countries is an important risk factor for the burden of neck pain. Interestingly, however, the neck pain burden was also high in some countries with a middle or low SDI. The unclear relationship between SDI and the burden of neck pain is likely due to differences in the risk factors (8, 9, 23, 25, 42) such as psychological factors (e.g., stress, anxiety, sleep problems) and biological factors (e.g., age, ethnicity, obesity, physical inactivity) among different regions and countries. In particular, a research approach is needed in countries such as the Philippines, which has a high neck pain burden with low-to-moderate SDI, and Australasia, which has a low neck pain burden with high SDI. Policymakers in individual countries should develop strategies by taking into account the neck pain burden of neighboring countries, the predicted neck pain burden of their country, and risk factors for neck pain.

The major limitation of GBD analysis is the availability of primary data. When data are not available for a specific region, results are modeled using DisMod-MR 2.1. Although data processing and modeling can improve the accuracy of data, this method has some fundamental problems (12). Furthermore, although data stability improved dramatically when data were adjusted to standard locations through bias mapping, collinearity between covariates in some of these models may have caused some instability in fixed effects between GBD data cycles. Although statistical modeling can be used to estimate uncertainty from stochastic variation in GBD, UIs around estimates in this way cannot fully cover the variation. Second, to estimate the severity distribution of neck pain, survey data were used, and these data could have been affected by recall bias due to the long follow-up period. Finally, because this study is an analysis of GBD based on the data obtained from various sources, it is not possible to know whether it is acute or chronic and what the severity is, which is important for diagnosis, treatment, and prognosis of neck pain.

As demonstrated in this study, neck pain is a public health problem worldwide, with great variation between countries. Age-standardized point prevalence, annual incidence, YLD of neck pain, and positive associations between SDI and burden of neck pain have not changed dramatically over the past three decades, but the burden of neck pain remains high among middle-aged men and women, particularly at risk. With aging global populations, policymakers must have accurate, up-to-date information about neck pain, and further research should be conducted to investigate risk factors for neck pain to reduce the future economic and social burdens of this condition.

## Data availability statement

Publicly available datasets were analyzed in this study. This data can be found here: Global Burden of Disease (GBD) Compare Viz Hub, https://vizhub.healthdata.org/gbd-compare.

## Ethics statement

The studies involving human participants were reviewed and approved by Institutional Review Board at Ewha Womans University Seoul Hospital. Written informed consent for participation was not required for this study in accordance with the national legislation and the institutional requirements.

## Author contributions

DS, JS, YC, and T-JS contributed to conception and design of the study. HL and T-JS organized the database. DS, HL, and T-JS performed the statistical analysis. DS and T-JS wrote the first draft of the manuscript. DS, JS, AK, LJ, LS, YC, and T-JS wrote sections of the manuscript. All authors contributed to manuscript revision, read, and approved the submitted version.

## Funding

This project was supported by a grant from the Basic Science Research Program through the National Research Foundation of Korea funded by the Ministry of Education (2021R1F1A1048113 to T-JS, 2021R1I1A1A01059868 to YC). The funding source had no role in the design, conduct, or reporting of the study.

## Conflict of interest

The authors declare that the research was conducted in the absence of any commercial or financial relationships that could be construed as a potential conflict of interest.

## Publisher's note

All claims expressed in this article are solely those of the authors and do not necessarily represent those of their affiliated organizations, or those of the publisher, the editors and the reviewers. Any product that may be evaluated in this article, or claim that may be made by its manufacturer, is not guaranteed or endorsed by the publisher.
