# Supplementary material for: Global, regional, and national neck pain burden in the general population, 1990–2019: An analysis of the global burden of disease study 2019
Source: Front Neurol. 2022 Sep 1;13:955367. doi: 10.3389/fneur.2022.955367 (PMC9477009; doi:10.3389/fneur.2022.955367)
Supplement: Supplementary file 1 [file Table_1.DOCX]

**S1 Table. Prevalence, Incidence, and YLDs of neck pain in counts and age-standardized rate for both sexes combined in 1990 and 2019, with percentage change between 1990 and 2019 by nation**

|  | **1990** | | **2019** | | **percentage change in  age-standardized rates  between 1990 and 2019 (%)** |
| --- | --- | --- | --- | --- | --- |
|  | **Counts (95% UI)** | **Age-standardized Rate (per 100k)** | **Counts (95% UI)** | **Age-standardized Rate (per 100k)** |  |
| **Prevalence** | | | | | |
| **Afghanistan** | 227118.92 (176296.71, 291044.17) | 2894.82 (2265.3, 3668.37) | 631667.6 (487329.83, 828692.55) | 2872.19 (2250.46, 3640.54) | -0.78 (-1.56, 0.03) |
| **Albania** | 37807.98 (29823.25, 48718.53) | 1409.85 (1120.03, 1782.9) | 48574.95 (38412.96, 61213.02) | 1413.26 (1123.65, 1787.45) | 0.24 (-0.09, 0.61) |
| **Algeria** | 464269.85 (366593.79, 596831.2) | 2876.96 (2251.79, 3642.93) | 1183532.62 (917995.77, 1523923.23) | 2865.24 (2243.84, 3627.85) | -0.41 (-0.63, -0.22) |
| **American Samoa** | 1092.89 (861.67, 1421.99) | 3205.88 (2555.63, 4078.59) | 1683.88 (1313.7, 2159.48) | 3224.65 (2567.96, 4104.52) | 0.59 (0.32, 0.85) |
| **Andorra** | 2142.03 (1699.14, 2730.38) | 3517.82 (2802.82, 4449.08) | 4021.95 (3190.25, 5122.91) | 3538.02 (2819.98, 4475.84) | 0.57 (0.37, 0.8) |
| **Angola** | 72630.87 (56687.61, 94808.31) | 1145.09 (903.33, 1462.67) | 214306.82 (166705.29, 278555.94) | 1153.79 (910.64, 1472.97) | 0.76 (0.41, 1.11) |
| **Antigua and Barbuda** | 725.9 (581.16, 925.47) | 1360.67 (1074.24, 1743.64) | 1432.25 (1111.58, 1856.14) | 1358.4 (1071.02, 1741.16) | -0.17 (-0.32, -0.04) |
| **Argentina** | 620761.16 (494636.36, 784889.69) | 1933.69 (1539.03, 2447.46) | 961521.46 (772058.85, 1212024.61) | 1934.02 (1539.41, 2447.84) | 0.02 (-0.02, 0.05) |
| **Armenia** | 44032.67 (34638.41, 56433.24) | 1418.1 (1129.6, 1791.38) | 52232.81 (41170.38, 65816.86) | 1418.07 (1129.43, 1791.93) | 0 (-0.14, 0.12) |
| **Australia** | 202540.88 (160349.49, 253949.16) | 1092.58 (862.77, 1384.65) | 343757.31 (271940.1, 428941.23) | 1092.71 (861.18, 1386.04) | 0.01 (-0.19, 0.16) |
| **Austria** | 330573.06 (265639.44, 414359.6) | 3582.07 (2856.68, 4529.78) | 418536.27 (337022.11, 525058.88) | 3561.49 (2841.38, 4510.21) | -0.57 (-0.9, -0.29) |
| **Azerbaijan** | 85188.89 (67142.12, 109636.56) | 1419.69 (1131.51, 1793.83) | 159087.07 (123371.69, 206985.04) | 1415.66 (1126.78, 1789.28) | -0.28 (-0.56, -0.08) |
| **Bahamas** | 2840.23 (2237.27, 3693.12) | 1360.5 (1074.27, 1743.64) | 5825.43 (4498.97, 7616.57) | 1359.62 (1072.92, 1742.62) | -0.06 (-0.14, -0.01) |
| **Bahrain** | 10338.05 (7934.79, 13853.01) | 2735.17 (2155.58, 3480.19) | 48399.07 (36018.79, 64265.67) | 2679.5 (2109.25, 3403.72) | -2.04 (-2.79, -1.31) |
| **Bangladesh** | 962708.24 (760225.28, 1244810.67) | 1404.54 (1112.91, 1777.15) | 2134057.9 (1695996.35, 2720376.55) | 1411.34 (1120.12, 1784.84) | 0.48 (0.1, 0.85) |
| **Barbados** | 3493.44 (2807, 4420.09) | 1361.84 (1074.6, 1745.69) | 5370.68 (4218.34, 6810.74) | 1359.41 (1072.53, 1742.61) | -0.18 (-0.32, -0.06) |
| **Belarus** | 168850.86 (133858.97, 213406.61) | 1421.56 (1134.22, 1796.4) | 182062.44 (143022.39, 228863.2) | 1419.78 (1131.88, 1793.85) | -0.13 (-0.26, -0.01) |
| **Belgium** | 355501.36 (283983.76, 446088.06) | 2980.74 (2350.18, 3764.79) | 435088.68 (349252.54, 545225.53) | 2976.21 (2347.31, 3759.91) | -0.15 (-0.33, -0.03) |
| **Belize** | 1598.23 (1262.61, 2064.13) | 1352.55 (1067.13, 1734.6) | 4879.36 (3802.9, 6352.48) | 1354.67 (1067.1, 1736.93) | 0.16 (0, 0.33) |
| **Benin** | 33128.74 (26257.68, 42829.81) | 1192.72 (943.44, 1531.79) | 91877.09 (72218.88, 119142.55) | 1189.41 (939.55, 1526.06) | -0.28 (-0.45, -0.09) |
| **Bermuda** | 913.99 (720.61, 1187.21) | 1359.79 (1074.05, 1742.78) | 1274.13 (996.27, 1613.77) | 1357.5 (1071.79, 1740.31) | -0.17 (-0.29, -0.05) |
| **Bhutan** | 5492.09 (4304.05, 7148.18) | 1409.54 (1120.06, 1784.14) | 9982.98 (7928.03, 12845.95) | 1407.25 (1117.22, 1780.14) | -0.16 (-0.4, -0.01) |
| **Bolivia (Plurinational State of)** | 59468.08 (46762.39, 77031.64) | 1358.21 (1071.38, 1741) | 142480.67 (111867.9, 184369.2) | 1356.35 (1069.68, 1739.38) | -0.14 (-0.22, -0.05) |
| **Bosnia and Herzegovina** | 65993.87 (51685.81, 85168.21) | 1415.65 (1127.68, 1787.84) | 65131.61 (51318.39, 82345.19) | 1414.35 (1125.12, 1787.88) | -0.09 (-0.25, 0.06) |
| **Botswana** | 9501.88 (7411.04, 12247.53) | 1154.76 (911.47, 1473.99) | 23666.97 (18487.81, 30745.42) | 1153.44 (909.92, 1471.93) | -0.11 (-0.29, 0.05) |
| **Brazil** | 2636831.69 (2064700.39, 3417584.63) | 2250.15 (1775.99, 2883.49) | 5484911.07 (4299843.55, 7072737.43) | 2241.89 (1770.55, 2870.64) | -0.37 (-0.63, -0.15) |
| **Brunei Darussalam** | 3112.01 (2412.82, 4083.38) | 1702 (1346.08, 2168.96) | 7758.66 (5975.5, 10162.95) | 1716.13 (1358.35, 2191.43) | 0.83 (0.59, 1.09) |
| **Bulgaria** | 151997.87 (119332.29, 191386.49) | 1414.16 (1124.85, 1787.56) | 142942.23 (114003.49, 178551.94) | 1413.74 (1124.79, 1787.03) | -0.03 (-0.16, 0.1) |
| **Burkina Faso** | 68722.45 (54207.62, 88330.96) | 1194.41 (945.21, 1534.45) | 168360.06 (132372.22, 217767.15) | 1195.42 (945.02, 1534.31) | 0.08 (-0.08, 0.28) |
| **Burundi** | 38659.21 (30275.24, 50106.62) | 1154.23 (911.07, 1474.25) | 84947.67 (66502.49, 110868.57) | 1141.83 (900.81, 1458.66) | -1.07 (-1.64, -0.56) |
| **Cabo Verde** | 2763.59 (2207.66, 3521.36) | 1209.45 (956.51, 1551.93) | 6186.67 (4881.03, 7986.62) | 1187.25 (936.55, 1519.41) | -1.84 (-2.67, -1.07) |
| **Cambodia** | 242725.64 (190511.13, 310199.95) | 3838.99 (3038.78, 4859.85) | 566394.42 (441476.26, 727369.65) | 3826.99 (3027.93, 4841.89) | -0.31 (-0.6, -0.05) |
| **Cameroon** | 76267.15 (60015.91, 98357.25) | 1187.18 (938.22, 1522.61) | 228336.02 (178769.57, 297589.11) | 1185.6 (936.36, 1520.41) | -0.13 (-0.22, -0.05) |
| **Canada** | 911521.02 (723775.85, 1151725.63) | 2942.54 (2327.5, 3729.95) | 1473471.27 (1164234.87, 1863698.87) | 2942.27 (2325.8, 3731.63) | -0.01 (-0.16, 0.12) |
| **Central African Republic** | 20131.51 (15683.95, 26128.09) | 1152.68 (909.22, 1471.51) | 40543.44 (31436.02, 52664.95) | 1151.24 (908.33, 1470.89) | -0.12 (-0.33, 0.08) |
| **Chad** | 43222.73 (34228.74, 55072.2) | 1192.98 (942.87, 1532.95) | 105217.31 (82891.85, 136299.36) | 1179.46 (931.61, 1516.03) | -1.13 (-1.61, -0.75) |
| **Chile** | 228302.23 (179716.6, 291095.96) | 1936.23 (1541.66, 2450.73) | 421422 (334500.31, 533606.67) | 1932.06 (1538.79, 2445.57) | -0.22 (-0.28, -0.16) |
| **China** | 37850795.7 (29872567.62, 48558259.49) | 3528.35 (2800.49, 4485.74) | 67966088.09 (53672884.64, 87161084.67) | 3571.96 (2868.2, 4517.63) | 1.24 (-4.53, 7.02) |
| **Colombia** | 332450.29 (261173.86, 432157.18) | 1356.31 (1069.45, 1738.87) | 704946.58 (555121.99, 905401.74) | 1359.54 (1072.22, 1743.25) | 0.24 (0.08, 0.41) |
| **Comoros** | 3355.37 (2643.69, 4302.16) | 1149.34 (907.16, 1468.85) | 7008.04 (5494.47, 8960.52) | 1149.42 (906.94, 1467.81) | 0.01 (-0.2, 0.23) |
| **Congo** | 17538.32 (13652.08, 22568.64) | 1154.24 (911.01, 1472.77) | 46685.13 (36266.59, 60774.39) | 1146.56 (904.89, 1464.77) | -0.67 (-1.03, -0.33) |
| **Cook Islands** | 489.92 (389.12, 625.58) | 3201.76 (2550.32, 4069.65) | 685.69 (537.41, 872.52) | 3230.91 (2572.34, 4114.62) | 0.91 (0.51, 1.28) |
| **Costa Rica** | 30973.64 (24533.22, 39971.76) | 1355.89 (1069.06, 1738.44) | 70670.6 (55464.45, 90892.45) | 1359.37 (1071.89, 1742.46) | 0.26 (0.1, 0.42) |
| **Croatia** | 83661.05 (65643.51, 106499.28) | 1416.93 (1128.88, 1789.49) | 85517.05 (67530.17, 106675.52) | 1414.07 (1124.83, 1787.88) | -0.2 (-0.41, -0.01) |
| **Cuba** | 145692.38 (115991.81, 187172.94) | 1355.21 (1068.31, 1737.88) | 209123.18 (163641.13, 269711.77) | 1356.18 (1069.52, 1739.84) | 0.07 (-0.01, 0.16) |
| **Cyprus** | 28903.24 (23158.23, 36359.08) | 3563.2 (2842.63, 4513.78) | 58168.21 (46566.43, 73739.87) | 3575.46 (2854.53, 4529.51) | 0.34 (0.14, 0.58) |
| **Czechia** | 172905.88 (137316.79, 218552.64) | 1416.73 (1128.21, 1790.24) | 212932.72 (169917.51, 266987.52) | 1412.7 (1123.31, 1786.2) | -0.28 (-0.49, -0.07) |
| **Côte d'Ivoire** | 83789.87 (65013.74, 109629.16) | 1173.79 (925.95, 1505.01) | 211395.66 (164605.41, 276609.29) | 1174.42 (926.89, 1506.92) | 0.05 (-0.06, 0.16) |
| **Democratic People's Republic of Korea** | 616204.39 (477272.79, 799054.13) | 3207.07 (2531.27, 4064.82) | 1009329.89 (791893, 1290615.28) | 3168.88 (2505.66, 4005.19) | -1.19 (-1.6, -0.79) |
| **Democratic Republic of the Congo** | 269423.11 (208929.16, 348307.21) | 1153.45 (909.9, 1473.56) | 653582.27 (509747.19, 850662.52) | 1149.82 (906.89, 1468.08) | -0.31 (-0.6, 0.01) |
| **Denmark** | 279214.73 (225795.77, 346340.07) | 4486.91 (3581.42, 5617.87) | 331443.43 (265950.35, 409741.7) | 4477.65 (3571.18, 5607.69) | -0.21 (-0.36, -0.08) |
| **Djibouti** | 3190.48 (2470.53, 4219.2) | 1141.07 (899.44, 1457.8) | 11196.53 (8700.71, 14625.36) | 1138.49 (898.08, 1454.09) | -0.23 (-0.5, 0.03) |
| **Dominica** | 861.66 (692.48, 1085.6) | 1360.96 (1073.72, 1746.02) | 1079.22 (854.51, 1380.29) | 1352.81 (1067.61, 1734.61) | -0.6 (-1.12, -0.12) |
| **Dominican Republic** | 68965.66 (54264.14, 89559.24) | 1356.17 (1068.84, 1738.73) | 141686.6 (111165.36, 183773.62) | 1355.24 (1068.95, 1738.25) | -0.07 (-0.19, 0.06) |
| **Ecuador** | 97044.2 (76408.71, 125645.36) | 1355.44 (1068.62, 1737.91) | 226273.29 (177970.39, 292397.66) | 1356.81 (1069.75, 1739.75) | 0.1 (0.03, 0.18) |
| **Egypt** | 1133159.37 (886452.47, 1461199.58) | 2863.86 (2243.33, 3627.15) | 2393781.49 (1858374.81, 3101067.22) | 2834.57 (2219.41, 3593.15) | -1.02 (-1.37, -0.72) |
| **El Salvador** | 50062.45 (39494.29, 64584.75) | 1359.46 (1071.93, 1742.51) | 82265.44 (65367.33, 105374) | 1366.08 (1077.13, 1750.05) | 0.49 (0.18, 0.81) |
| **Equatorial Guinea** | 3106.28 (2422.96, 4001.26) | 1157.7 (914.02, 1477.87) | 10251.62 (7986.47, 13476.91) | 1156.26 (912.01, 1475.03) | -0.12 (-0.55, 0.23) |
| **Eritrea** | 20195.24 (15667.51, 26439.43) | 1156.28 (912.04, 1475.57) | 52854.48 (41079.05, 69167.78) | 1152.68 (909.12, 1471.25) | -0.31 (-0.47, -0.15) |
| **Estonia** | 26071.58 (20579.82, 33117.5) | 1421.88 (1134.78, 1796.54) | 25728.22 (20524.94, 31904.88) | 1416.72 (1128.44, 1790.31) | -0.36 (-0.61, -0.09) |
| **Eswatini** | 5363.4 (4205.99, 6963.72) | 1154.8 (911.48, 1475.18) | 9888.57 (7741.61, 12846.63) | 1157.12 (912.97, 1476.91) | 0.2 (-0.08, 0.54) |
| **Ethiopia** | 412464.85 (319014.21, 537889.69) | 1355.04 (1071.02, 1746.08) | 919652.79 (714170.7, 1203382.47) | 1355.36 (1070.91, 1746.05) | 0.02 (-0.1, 0.13) |
| **Fiji** | 18101.64 (14189.51, 23535.84) | 3219.29 (2563.75, 4095.94) | 28561.95 (22355.4, 36770.31) | 3220.5 (2567.51, 4097.13) | 0.04 (-0.1, 0.21) |
| **Finland** | 259183.3 (209899.05, 323383.49) | 4323.11 (3514.07, 5377.42) | 311565.66 (251892.11, 385070.2) | 4305.96 (3419.79, 5365.8) | -0.4 (-6.71, 6.14) |
| **France** | 1851421.11 (1501380.91, 2335092.85) | 2793.95 (2252.49, 3530.98) | 2346146.51 (1899943.95, 2917064.1) | 2792.68 (2250.97, 3529.56) | -0.05 (-0.19, 0.07) |
| **Gabon** | 7876.94 (6158.64, 10064.67) | 1150.68 (907.33, 1468.9) | 16605.48 (12953.49, 21439.75) | 1149.99 (907.02, 1469.01) | -0.06 (-0.41, 0.26) |
| **Gambia** | 6710.25 (5260.03, 8730.92) | 1172.31 (926.04, 1505.18) | 17399.13 (13690.71, 22595.67) | 1184.49 (935.71, 1520.07) | 1.04 (0.7, 1.46) |
| **Georgia** | 84188.65 (66520.56, 107065.18) | 1420.78 (1132.87, 1795.59) | 66564.09 (52601.92, 83212.83) | 1417.51 (1129.28, 1791.04) | -0.23 (-0.42, -0.06) |
| **Germany** | 3517201.6 (2818587.13, 4433167.91) | 3576.05 (2851.89, 4522.22) | 4063496.56 (3257233.88, 5073203.02) | 3552.8 (2833.46, 4497.03) | -0.65 (-1, -0.34) |
| **Ghana** | 112395.47 (88375.72, 145584.92) | 1186.56 (937.69, 1521.34) | 286192.99 (224414.59, 370237.34) | 1195.4 (944.91, 1533.1) | 0.75 (0.49, 1.05) |
| **Greece** | 477573.57 (382715.26, 601448.33) | 3889.16 (3120.45, 4903.67) | 547630.87 (441273.51, 687677.97) | 3888.59 (3120.39, 4903.01) | -0.01 (-0.07, 0.03) |
| **Greenland** | 1472.32 (1146.02, 1906.81) | 2880.37 (2278.19, 3648.67) | 1968.23 (1514.51, 2548.27) | 2903.39 (2294.02, 3687.05) | 0.8 (0.2, 1.31) |
| **Grenada** | 909.55 (725.57, 1148.11) | 1360.58 (1073.89, 1744.8) | 1556.83 (1212.09, 2028.71) | 1354.4 (1069.96, 1736.91) | -0.45 (-0.78, -0.15) |
| **Guam** | 3661.9 (2873.82, 4757.44) | 3200.01 (2552.36, 4069.72) | 5836.87 (4575.79, 7500.13) | 3211.17 (2560.47, 4083.69) | 0.35 (0.18, 0.53) |
| **Guatemala** | 68960.26 (54010.54, 89406.37) | 1355.67 (1068.54, 1738.11) | 193547.4 (152859.54, 250135.78) | 1362.52 (1073.71, 1746.28) | 0.51 (0.19, 0.83) |
| **Guinea** | 48736.88 (38620.04, 62424.57) | 1187.41 (938.96, 1525.44) | 94215.96 (74352.37, 121765.83) | 1188.01 (939.36, 1526.47) | 0.05 (-0.05, 0.16) |
| **Guinea-Bissau** | 7130.25 (5626.99, 9236.05) | 1192.97 (943.15, 1532.92) | 14564.59 (11358.16, 19078.51) | 1193.33 (943.24, 1531.59) | 0.03 (-0.14, 0.22) |
| **Guyana** | 7445.67 (5860.48, 9723.79) | 1356.4 (1069.78, 1739.04) | 10013.5 (7773.4, 13045.18) | 1357.73 (1071.52, 1740.38) | 0.1 (0.03, 0.17) |
| **Haiti** | 59710.79 (46934.38, 77271.21) | 1357.15 (1071.32, 1739.28) | 135505.62 (105717.58, 176434.83) | 1359.49 (1071.68, 1742.67) | 0.17 (0, 0.36) |
| **Honduras** | 38892.21 (30536.37, 50442.11) | 1356.17 (1068.96, 1738.76) | 106838.95 (84149.91, 138395.57) | 1359.53 (1071.76, 1742.64) | 0.25 (0.1, 0.41) |
| **Hungary** | 180059.34 (142137.19, 227505.47) | 1417.59 (1129.18, 1791.1) | 195967.34 (156458.41, 245104.21) | 1415.93 (1127.21, 1789.56) | -0.12 (-0.23, -0.01) |
| **Iceland** | 9339.65 (7478.09, 11727.89) | 3559.06 (2838.59, 4505.28) | 14643.62 (11726.83, 18269.27) | 3550.69 (2830.89, 4492.58) | -0.24 (-0.34, -0.13) |
| **India** | 10601051.72 (8270456.67, 13741750.66) | 1651.79 (1322.59, 2090.93) | 22108450.54 (17438336.63, 28372167.23) | 1655.5 (1326.3, 2094.8) | 0.22 (0.07, 0.42) |
| **Indonesia** | 5992751.13 (4658363.76, 7779504.17) | 4306.84 (3413.65, 5479.71) | 11613902.51 (9014291.63, 15168806.33) | 4302.58 (3410.29, 5473.63) | -0.1 (-0.18, -0.02) |
| **Iran (Islamic Republic of)** | 1552228.49 (1226999.45, 2008889) | 4246.34 (3359.69, 5396.66) | 3880233.49 (3041905.85, 5013339.51) | 4288.71 (3391.67, 5448.37) | 1 (0.63, 1.42) |
| **Iraq** | 301819.43 (236897.22, 387669.77) | 2863.66 (2244.04, 3626.09) | 952368.4 (743005.12, 1235122.43) | 2862.68 (2242.88, 3626.11) | -0.03 (-0.1, 0.04) |
| **Ireland** | 131208.68 (105492.78, 163853.83) | 3564.2 (2843.13, 4512.92) | 210752.83 (168860.98, 266247.67) | 3567.15 (2847.18, 4518.79) | 0.08 (-0.08, 0.22) |
| **Israel** | 168995.93 (135513.97, 212007.16) | 3578.52 (2856.6, 4531.77) | 346167.74 (277495.59, 432451) | 3567.14 (2846.21, 4518.55) | -0.32 (-0.42, -0.21) |
| **Italy** | 2664062.76 (2145774.13, 3370582.72) | 3861.7 (3087.74, 4893.29) | 3191062.57 (2590034.4, 3965564.7) | 3848.98 (3080.42, 4874.44) | -0.33 (-0.47, -0.21) |
| **Jamaica** | 25057.55 (20108.23, 32032.72) | 1357.54 (1071.04, 1740.3) | 40740.76 (32157.38, 52531.81) | 1356.19 (1069.95, 1738.48) | -0.1 (-0.21, 0) |
| **Japan** | 3093832.16 (2411622.28, 3938787.58) | 1962.85 (1550.85, 2486.9) | 3837942.26 (3069545.24, 4767863.35) | 1954.59 (1544.42, 2477.85) | -0.42 (-0.62, -0.26) |
| **Jordan** | 60850.43 (47358.12, 79236.15) | 2843.77 (2229.64, 3598.11) | 274036.04 (211610.64, 355008.5) | 2815.78 (2209.23, 3566.78) | -0.98 (-1.28, -0.74) |
| **Kazakhstan** | 207198.91 (164539.66, 264202.37) | 1421.26 (1133.94, 1796.01) | 270770.95 (212999.25, 347368.33) | 1419.27 (1130.93, 1792.97) | -0.14 (-0.31, 0) |
| **Kenya** | 175822.84 (136390.11, 228451.23) | 1358.01 (1072.52, 1749.04) | 483045.99 (374516.45, 633281.24) | 1358.74 (1073.26, 1750.23) | 0.05 (-0.05, 0.18) |
| **Kiribati** | 1694.89 (1335.26, 2199.4) | 3239.18 (2578.65, 4126.94) | 3096.76 (2433.37, 4004.91) | 3248.63 (2582.55, 4140.78) | 0.29 (0.15, 0.43) |
| **Kuwait** | 28310.71 (21661.57, 37672.95) | 2099.69 (1652.77, 2690.75) | 108139.5 (81741.17, 145244.61) | 2167.49 (1705.38, 2779.37) | 3.23 (2.35, 4.1) |
| **Kyrgyzstan** | 49005.56 (38910.58, 62783.97) | 1419.33 (1131.59, 1792.69) | 82156.79 (64409.28, 106150.16) | 1417.14 (1128.49, 1790.48) | -0.15 (-0.37, 0) |
| **Lao People's Democratic Republic** | 104362.67 (81797.85, 133009.26) | 3815.46 (3024.74, 4817.92) | 228919.35 (178117.51, 294865.87) | 3807.7 (3020.22, 4805.72) | -0.2 (-0.35, -0.06) |
| **Latvia** | 44902.05 (35511.77, 56985.9) | 1422.17 (1135.09, 1796.82) | 38384.16 (30466.39, 47765.94) | 1418.74 (1130.76, 1792.48) | -0.24 (-0.42, -0.05) |
| **Lebanon** | 75402.82 (58621.35, 96506.86) | 2886.85 (2261.19, 3652.37) | 156590.92 (123054.69, 199776.44) | 2919.54 (2279.69, 3701.04) | 1.13 (0.69, 1.62) |
| **Lesotho** | 14343.31 (11247.75, 18364.62) | 1151.95 (908.54, 1471.47) | 19946.64 (15563.9, 25728.7) | 1154.83 (910.69, 1473.02) | 0.25 (0.01, 0.53) |
| **Liberia** | 15750.71 (12464.95, 20091.01) | 1177.97 (931.55, 1512.06) | 40238.31 (31256.99, 52375.7) | 1178.14 (930.23, 1512.55) | 0.01 (-0.14, 0.17) |
| **Libya** | 72247.92 (56703.56, 93290.07) | 2797.15 (2197.7, 3544.14) | 201927.94 (154757.95, 264591.68) | 2848.64 (2231.14, 3608.35) | 1.84 (1.43, 2.32) |
| **Lithuania** | 58953.95 (46731.24, 74827.59) | 1420.75 (1133.05, 1795.43) | 55822.65 (44233.19, 69556.65) | 1418.86 (1130.83, 1792.61) | -0.13 (-0.25, -0.01) |
| **Luxembourg** | 16330.21 (13034.07, 20553.47) | 3569.37 (2846.68, 4518.1) | 27869.29 (22276.53, 35085.47) | 3550.95 (2831.28, 4490.86) | -0.52 (-0.84, -0.26) |
| **Madagascar** | 84109.8 (65517.62, 108581.12) | 1146.98 (905.24, 1466.78) | 207745.22 (161530.2, 270337.19) | 1148.21 (905.95, 1467.42) | 0.11 (0, 0.25) |
| **Malawi** | 66403.56 (52163.65, 85992.15) | 1150.54 (908.02, 1469.5) | 133923.22 (104915.65, 174683.27) | 1151.95 (909.07, 1471.06) | 0.12 (0, 0.26) |
| **Malaysia** | 349191.81 (284590.86, 437127.02) | 2696.41 (2210.62, 3292.77) | 946321.09 (741481.9, 1208995.25) | 3013.6 (2369.85, 3818.7) | 11.76 (2.7, 23.08) |
| **Maldives** | 4920.62 (3830.52, 6333.14) | 3780.86 (3001.87, 4772.63) | 18739.8 (14540.98, 24563.7) | 3758.82 (2981.03, 4736.49) | -0.58 (-1.22, 0.04) |
| **Mali** | 65194.76 (51173.35, 83886.04) | 1186.32 (937.95, 1522.71) | 152648.25 (120276.12, 197646.8) | 1182.2 (934.02, 1518.65) | -0.35 (-0.48, -0.21) |
| **Malta** | 14674.73 (11656.65, 18479.99) | 3577.6 (2854.5, 4528.92) | 20682.84 (16680.62, 25859.69) | 3552.92 (2832.71, 4495.03) | -0.69 (-0.91, -0.44) |
| **Marshall Islands** | 827.76 (652.77, 1072.17) | 3209.58 (2559.02, 4083.1) | 1576.47 (1232.16, 2040.72) | 3213.53 (2558.44, 4085.89) | 0.12 (-0.12, 0.37) |
| **Mauritania** | 15683.97 (12431.38, 20107.27) | 1187.72 (938.23, 1523.28) | 33578.16 (26450.82, 43032.39) | 1187.47 (937.9, 1524.79) | -0.02 (-0.17, 0.1) |
| **Mauritius** | 36048.81 (28380.79, 46129.52) | 3813.01 (3021.89, 4811.65) | 62495.78 (48792.06, 80555.45) | 3809.56 (3020.18, 4806.1) | -0.09 (-0.17, -0.02) |
| **Mexico** | 949903.27 (743220.3, 1217224.07) | 1594.04 (1257.94, 2056.6) | 2043925.73 (1595139.23, 2643730.2) | 1595.89 (1258.92, 2058.85) | 0.12 (0.04, 0.2) |
| **Micronesia (Federated States of)** | 2102.53 (1672.5, 2699.34) | 3212.72 (2560.83, 4084.18) | 2905.37 (2255.47, 3759.23) | 3223.66 (2569.15, 4101.51) | 0.34 (0.17, 0.51) |
| **Monaco** | 1505.06 (1211.08, 1873.04) | 3579.5 (2857.07, 4529.75) | 1877.7 (1504.07, 2342.57) | 3570.45 (2850.93, 4519.41) | -0.25 (-0.44, -0.07) |
| **Mongolia** | 20066.5 (15857.93, 25971.63) | 1414.23 (1124.79, 1787.71) | 46075.9 (35463.28, 60600.78) | 1417.25 (1128.74, 1790.01) | 0.21 (0.02, 0.4) |
| **Montenegro** | 9125.19 (7240.1, 11654.49) | 1415.37 (1126.76, 1788.61) | 11383.42 (8970.44, 14276.25) | 1414.09 (1124.86, 1787.98) | -0.09 (-0.2, 0.01) |
| **Morocco** | 516188.42 (404497.09, 663898.47) | 2880.34 (2255.35, 3646.92) | 1049720.16 (809891.5, 1345814.6) | 2874.25 (2251.27, 3637.42) | -0.21 (-0.32, -0.09) |
| **Mozambique** | 97348.5 (75899.23, 125237.07) | 1152.24 (909.65, 1472.46) | 202414.95 (158564.09, 263242.49) | 1154.45 (910.88, 1473.5) | 0.19 (0.02, 0.37) |
| **Myanmar** | 1143099.62 (895442.5, 1453592.35) | 3814.01 (3023.42, 4814.6) | 2066815.43 (1607921.11, 2652897.88) | 3826.48 (3029.62, 4836.68) | 0.33 (0.1, 0.56) |
| **Namibia** | 10764.08 (8463.64, 13793.59) | 1151.4 (908.75, 1470.22) | 22000.22 (17327.34, 28397.9) | 1155.18 (911.62, 1473.55) | 0.33 (0.16, 0.53) |
| **Nauru** | 214.3 (167.04, 279.15) | 3201 (2551.25, 4071.35) | 248.16 (191.71, 323.09) | 3239.91 (2575.14, 4127.2) | 1.22 (0.68, 1.73) |
| **Nepal** | 186546.51 (146212.95, 239566.4) | 1410.41 (1119.35, 1783.4) | 377332.4 (299032.75, 480758.36) | 1417.5 (1127.15, 1790.66) | 0.5 (0.11, 0.88) |
| **Netherlands** | 557756.04 (446633.75, 695904.93) | 3229.4 (2592.51, 4057.71) | 715545.97 (566915.26, 894041.49) | 3223.44 (2587.21, 4055.52) | -0.18 (-0.44, 0.02) |
| **New Zealand** | 42705.55 (33299.75, 55446.72) | 1163.71 (901.16, 1519.66) | 50116.13 (40438.15, 61326.02) | 871.01 (702.23, 1082.49) | -25.15 (-32.54, -17.86) |
| **Nicaragua** | 30842.31 (24194.64, 40440.74) | 1358.47 (1072.01, 1740.91) | 77621.47 (60879.55, 100601.26) | 1360.11 (1072.71, 1743.94) | 0.12 (-0.03, 0.27) |
| **Niger** | 53089.96 (41535.27, 69088.64) | 1178.92 (931.22, 1513.14) | 146023.98 (115032.91, 188844.68) | 1189.39 (939.11, 1526.6) | 0.89 (0.57, 1.26) |
| **Nigeria** | 847233.75 (665088.22, 1096487.59) | 1414.49 (1126.63, 1803.62) | 2050438.67 (1598329.13, 2665491.67) | 1492.02 (1184.41, 1928.4) | 5.48 (0.86, 10.27) |
| **Niue** | 66.3 (52.96, 83.82) | 3231.52 (2573.44, 4114.78) | 62.11 (48.78, 78.92) | 3223.59 (2568.6, 4101.83) | -0.25 (-0.44, -0.08) |
| **North Macedonia** | 28791.07 (22768.86, 36757.89) | 1412.64 (1123.24, 1785.3) | 39881.51 (31447.54, 50516.65) | 1410.91 (1121.31, 1784.43) | -0.12 (-0.26, 0) |
| **Northern Mariana Islands** | 1212.81 (923.83, 1614.72) | 3150.56 (2514.52, 3999.69) | 1721.99 (1286.32, 2268.91) | 3204.65 (2555.82, 4077.5) | 1.72 (0.91, 2.54) |
| **Norway** | 163098.86 (131846.29, 204872.77) | 3238.82 (2571.94, 4127.02) | 175838.26 (140264.22, 218890.05) | 2659.99 (2099.64, 3366.72) | -17.87 (-23.67, -11.22) |
| **Oman** | 33441.78 (25887.29, 44117.48) | 2717.22 (2142.21, 3454.94) | 110294.51 (84179.95, 148639.62) | 2675.93 (2111.44, 3394.49) | -1.52 (-1.98, -1.13) |
| **Pakistan** | 1218553.91 (959571.86, 1557417.29) | 1649.59 (1319.59, 2089.67) | 2681714.11 (2095887.47, 3467203.93) | 1653.86 (1324.51, 2093.22) | 0.26 (0.1, 0.42) |
| **Palau** | 417.07 (331.52, 536.85) | 3215.88 (2562.19, 4090.19) | 735.55 (563.05, 961.19) | 3193.29 (2545.45, 4054.25) | -0.7 (-1.13, -0.32) |
| **Palestine** | 33197.26 (26161.78, 42786.26) | 2921.28 (2280.02, 3709.01) | 101781.89 (79243.33, 131698.79) | 2867.99 (2248.44, 3630.16) | -1.82 (-2.49, -1.25) |
| **Panama** | 25377.56 (20043.58, 32864.03) | 1353.29 (1066.95, 1735.45) | 57022.43 (44909.36, 73354.63) | 1355.1 (1068.51, 1737.99) | 0.13 (0.05, 0.22) |
| **Papua New Guinea** | 88758.17 (69877.49, 115466.34) | 3205.88 (2554.15, 4076.29) | 237728.12 (186200.05, 306868.6) | 3205.49 (2552.29, 4075.13) | -0.01 (-0.15, 0.11) |
| **Paraguay** | 46733.73 (36788.81, 59826.92) | 1654.31 (1307.91, 2124.39) | 106534.51 (83995.61, 138262.45) | 1654 (1307.58, 2124.41) | -0.02 (-0.05, 0.01) |
| **Peru** | 213095.53 (167678.76, 275862.89) | 1355.79 (1069.16, 1738.17) | 463757.93 (364646.12, 600153.18) | 1356.78 (1069.37, 1739.81) | 0.07 (0, 0.15) |
| **Philippines** | 2313176.68 (1822198.12, 2979783.23) | 5335.26 (4273.72, 6746.19) | 5251343.81 (4135080.41, 6727792.95) | 5333.53 (4269.74, 6740.66) | -0.03 (-0.13, 0.08) |
| **Poland** | 696526.69 (554256.22, 882086.67) | 1662.38 (1332.83, 2100.33) | 880078.95 (700711.32, 1099178.39) | 1659.71 (1330.39, 2098.09) | -0.16 (-0.29, -0.05) |
| **Portugal** | 416107.94 (333658.67, 520195.81) | 3588.74 (2863.66, 4540.67) | 523766.6 (422327.99, 653441.49) | 3583.74 (2860.44, 4535.81) | -0.14 (-0.22, -0.07) |
| **Puerto Rico** | 49002.49 (38908.74, 62551.44) | 1359.92 (1072.21, 1743.08) | 65638.75 (51935.04, 82042.97) | 1360.15 (1072.86, 1743.38) | 0.02 (-0.04, 0.08) |
| **Qatar** | 9424.01 (7074.38, 12780.51) | 2586.36 (2038.67, 3263.06) | 74937.78 (56460.52, 101342.43) | 2513.14 (1977.79, 3177.86) | -2.83 (-3.78, -1.94) |
| **Republic of Korea** | 698510.34 (547488.59, 900822.7) | 1735.86 (1372.76, 2219.94) | 1330805.2 (1035486.92, 1716308.63) | 1719.45 (1361.87, 2196.17) | -0.95 (-1.26, -0.59) |
| **Republic of Moldova** | 64509.65 (51227.5, 82496.86) | 1420.32 (1132.1, 1794.41) | 69890.05 (55026.08, 88314.05) | 1417.59 (1129.34, 1790.64) | -0.19 (-0.33, -0.04) |
| **Romania** | 369460.01 (289460.1, 468685.3) | 1414.98 (1126.03, 1788.02) | 382098.54 (303219.81, 479198.52) | 1414.01 (1124.97, 1787.31) | -0.07 (-0.16, 0.01) |
| **Russian Federation** | 2851362.2 (2250944.74, 3640039.05) | 1668.46 (1338.79, 2108.28) | 3222675.81 (2554282.69, 4077699.4) | 1665.48 (1335.94, 2103.82) | -0.18 (-0.36, -0.03) |
| **Rwanda** | 49157.14 (38489.36, 63702.85) | 1155.42 (911.7, 1475.17) | 105646.25 (82379.12, 136732.57) | 1156.57 (912.44, 1475.65) | 0.1 (-0.01, 0.24) |
| **Saint Kitts and Nevis** | 461.28 (367.28, 583.47) | 1359.55 (1072.65, 1743.13) | 965.02 (739.49, 1261.25) | 1354.72 (1069.21, 1737.11) | -0.36 (-0.61, -0.11) |
| **Saint Lucia** | 1359.66 (1081.36, 1737.24) | 1360.35 (1073.42, 1743.65) | 2873.69 (2248.19, 3725.52) | 1356.22 (1070.08, 1739.02) | -0.3 (-0.51, -0.1) |
| **Saint Vincent and the Grenadines** | 1081.15 (862.41, 1383.12) | 1358.57 (1072.2, 1742.5) | 1755.79 (1372.27, 2261.15) | 1352.32 (1066.38, 1734.12) | -0.46 (-0.81, -0.14) |
| **Samoa** | 3563.88 (2821.19, 4576.91) | 3216.29 (2563.52, 4091.04) | 5575.57 (4410.53, 7133.19) | 3211.52 (2560.54, 4085.25) | -0.15 (-0.3, 0) |
| **San Marino** | 964.93 (775.98, 1205.34) | 3559.83 (2837.58, 4504.24) | 1526.26 (1234.51, 1908.47) | 3587.11 (2864.85, 4542.46) | 0.77 (0.38, 1.23) |
| **Sao Tome and Principe** | 901.24 (714.84, 1154.37) | 1189.64 (939.53, 1527.77) | 1857.67 (1454.57, 2392.26) | 1184.2 (934.59, 1517.89) | -0.46 (-0.71, -0.24) |
| **Saudi Arabia** | 276459.12 (215080.45, 361573.42) | 2724.05 (2144.74, 3458.4) | 1000012.26 (761517.31, 1326632.79) | 2727.84 (2143.3, 3464.93) | 0.14 (-0.18, 0.41) |
| **Senegal** | 53792.8 (42508.48, 69273.4) | 1186.18 (937.14, 1523.38) | 124942.82 (98288.47, 160653.99) | 1189.05 (939.12, 1525.97) | 0.24 (0.11, 0.4) |
| **Serbia** | 153915.39 (120215.2, 197197) | 1413.92 (1124.36, 1787.5) | 166086.13 (131329.34, 207419.69) | 1413.13 (1123.84, 1786.56) | -0.06 (-0.11, 0) |
| **Seychelles** | 2287.9 (1824.27, 2856.13) | 3808.97 (3016.78, 4811.73) | 4555.33 (3537.63, 5885.57) | 3792.33 (3009.03, 4785.35) | -0.44 (-0.75, -0.14) |
| **Sierra Leone** | 29145.46 (23100.6, 37354.15) | 1183.6 (935.18, 1519.44) | 65535.58 (51440.87, 85147.19) | 1181.4 (932.95, 1516.23) | -0.19 (-0.31, -0.05) |
| **Singapore** | 51960.05 (40553.16, 67365.89) | 1732.64 (1371.58, 2216.91) | 133543.53 (104057.67, 173466) | 1713.45 (1357.62, 2184.66) | -1.11 (-1.55, -0.73) |
| **Slovakia** | 80681.82 (64143.24, 101997.04) | 1416.78 (1128.18, 1790.26) | 105354.96 (83120.44, 132720.97) | 1414.52 (1125.51, 1788.3) | -0.16 (-0.3, -0.01) |
| **Slovenia** | 32483.91 (25607.61, 41254.57) | 1417.1 (1129.1, 1789.22) | 42114.09 (33269.72, 52572.09) | 1411.48 (1121.9, 1785.25) | -0.4 (-0.72, -0.06) |
| **Solomon Islands** | 6500.22 (5104.3, 8471.68) | 3195.16 (2541.83, 4061.02) | 15292.49 (11962.74, 19816.96) | 3216.88 (2561.62, 4091.65) | 0.68 (0.36, 1) |
| **Somalia** | 49776.39 (38704.37, 64447.5) | 1149.88 (907.48, 1469.01) | 136325.17 (106255.88, 177470.55) | 1153.31 (909.68, 1471.21) | 0.3 (0.06, 0.57) |
| **South Africa** | 380182.53 (297569.39, 492488.98) | 1365.66 (1077.68, 1758.11) | 738007.79 (577679.99, 964600) | 1364.89 (1077.41, 1757.51) | -0.06 (-0.13, 0.01) |
| **South Sudan** | 40569.58 (31902.64, 52844.07) | 1134.38 (894.88, 1447.83) | 68673 (53168.95, 88893.19) | 1145.76 (904.76, 1466.07) | 1 (0.51, 1.51) |
| **Spain** | 1199418.46 (953249.98, 1507545.29) | 2706.78 (2147.63, 3418.32) | 1676583.36 (1338561.16, 2106757.31) | 2698.63 (2139.86, 3407.69) | -0.3 (-0.43, -0.18) |
| **Sri Lanka** | 544386.68 (425140.44, 701727.51) | 3804.27 (3019.78, 4805.34) | 955133.46 (747902.27, 1223619.19) | 3819.22 (3025.64, 4824.38) | 0.39 (0.06, 0.75) |
| **Sudan** | 357950.5 (281397.02, 459806.2) | 2861.42 (2241.84, 3621.3) | 804491.3 (626528.33, 1044812.19) | 2852.11 (2234.14, 3611.63) | -0.33 (-0.53, -0.12) |
| **Suriname** | 4238.51 (3318.36, 5499.03) | 1356.65 (1070.09, 1739.88) | 8418 (6555.97, 10886) | 1357.79 (1071.74, 1740.38) | 0.08 (-0.04, 0.23) |
| **Sweden** | 455160.4 (369715.65, 566321.35) | 4330.89 (3464.19, 5479.89) | 559713.54 (454555.7, 691894.69) | 4344.75 (3470.51, 5510.23) | 0.32 (-0.14, 0.91) |
| **Switzerland** | 295504.63 (237275.1, 368785.63) | 3566.71 (2845.23, 4515.24) | 409350.1 (330550.42, 514252.52) | 3554.84 (2835.05, 4498.12) | -0.33 (-0.54, -0.17) |
| **Syrian Arab Republic** | 209002.03 (164194.08, 270202.6) | 2851.61 (2233.4, 3612.39) | 414330.14 (315819.3, 538815.02) | 2889.58 (2265.08, 3663.18) | 1.33 (0.98, 1.78) |
| **Taiwan (Province of China)** | 610935.24 (476487.43, 774217.56) | 3172.91 (2497.54, 4008.72) | 994190.66 (859182.4, 1155693.99) | 2925.52 (2547, 3374.16) | -7.8 (-20.19, 7) |
| **Tajikistan** | 49836.88 (39401.51, 64194.52) | 1415.11 (1126.39, 1786.92) | 108357.42 (83708.16, 141718.01) | 1413.06 (1123.57, 1786.38) | -0.14 (-0.43, 0.06) |
| **Thailand** | 1836489.02 (1431348.98, 2360413.74) | 3813.87 (3023.24, 4815.44) | 3628842.05 (2827755.63, 4683403.14) | 3815.15 (3023.7, 4817.03) | 0.03 (0, 0.07) |
| **Timor-Leste** | 18884.14 (14614.9, 24443.48) | 3797.43 (3015.41, 4795.57) | 36999.59 (29253.64, 46920.56) | 3799.3 (3016.13, 4798.6) | 0.05 (-0.05, 0.15) |
| **Togo** | 24509.93 (19217.2, 31912.53) | 1193.44 (943.36, 1533.84) | 68017.69 (53004.34, 88068.76) | 1194.83 (944.65, 1532.97) | 0.12 (-0.14, 0.41) |
| **Tokelau** | 42.14 (33.7, 53.29) | 3252.26 (2588.5, 4148.48) | 42.82 (33.75, 54.88) | 3214.81 (2561.01, 4088.24) | -1.15 (-1.63, -0.62) |
| **Tonga** | 2179.46 (1723.84, 2784.72) | 3233.54 (2573.24, 4119.77) | 2793.22 (2219.49, 3555.51) | 3231.52 (2573.96, 4116.18) | -0.06 (-0.23, 0.1) |
| **Trinidad and Tobago** | 13639.64 (10780.81, 17602.14) | 1356.17 (1069.92, 1739.03) | 23199.16 (18190.8, 29598.22) | 1355 (1069.18, 1737.67) | -0.09 (-0.14, -0.03) |
| **Tunisia** | 177592.82 (139311.23, 228823.5) | 2864.99 (2242.03, 3626.73) | 381407.66 (293932.21, 486931.49) | 2883.14 (2258.73, 3648.4) | 0.63 (0.44, 0.87) |
| **Turkey** | 1307041.67 (1019150.11, 1679148.85) | 2875.69 (2253.21, 3640.54) | 2696086.41 (2091023.99, 3434490.77) | 2875.01 (2252.61, 3640.76) | -0.02 (-0.09, 0.04) |
| **Turkmenistan** | 35988.34 (28463.27, 46610.95) | 1418.22 (1130.08, 1791.01) | 68018.63 (53001.79, 87364.1) | 1414 (1125.69, 1787.01) | -0.3 (-0.58, -0.06) |
| **Tuvalu** | 258.84 (205.22, 331.1) | 3262.57 (2596.8, 4165.65) | 358.34 (283.08, 458.22) | 3218.95 (2563.93, 4093.39) | -1.34 (-1.93, -0.71) |
| **Uganda** | 111391.85 (87079.68, 144199.41) | 1148.32 (906.07, 1467.76) | 273492.62 (213633.04, 357372.04) | 1154.26 (910.94, 1473.04) | 0.52 (0.26, 0.8) |
| **Ukraine** | 1044669.55 (829416.86, 1326416.77) | 1668.05 (1338.21, 2108.49) | 1008946.37 (801085.71, 1272548.03) | 1666.02 (1336.33, 2105.72) | -0.12 (-0.23, -0.03) |
| **United Arab Emirates** | 36825.77 (27568.56, 50309.25) | 2593.86 (2045.33, 3282.61) | 301532.91 (221561.28, 413691.4) | 2536.19 (2000.62, 3197.87) | -2.22 (-3.19, -1.42) |
| **United Kingdom** | 3128216.43 (2533688.51, 3851564.93) | 4485.59 (3587.67, 5642.01) | 3770899.32 (3029424.22, 4701190.96) | 4501.34 (3591.7, 5675.23) | 0.35 (-4.98, 6.48) |
| **United Republic of Tanzania** | 178511.76 (139566.09, 229755.66) | 1150.15 (907.7, 1470.19) | 427842.08 (335539.88, 553424.37) | 1150.28 (907.72, 1470.1) | 0.01 (-0.02, 0.05) |
| **United States of America** | 12348098.51 (9975794.97, 15461040.49) | 4325.43 (3473.66, 5461.38) | 21184349.12 (17566737.15, 25306220.33) | 5123.29 (4268.35, 6170.35) | 18.45 (10.01, 27.57) |
| **United States Virgin Islands** | 1407.12 (1085.15, 1845.56) | 1359.64 (1073.21, 1742.33) | 1881.07 (1472.38, 2382.92) | 1360.93 (1074.06, 1743.93) | 0.1 (0.02, 0.18) |
| **Uruguay** | 65566.09 (52495.31, 81928.75) | 1935.11 (1539.94, 2449.45) | 80845.3 (64963.26, 100507.12) | 1936.81 (1541.15, 2451.85) | 0.09 (0.05, 0.12) |
| **Uzbekistan** | 204159.97 (161490.44, 263550.3) | 1417.39 (1129.17, 1789.71) | 427623.06 (328751.42, 558242.58) | 1416.35 (1127.41, 1790.24) | -0.07 (-0.26, 0.07) |
| **Vanuatu** | 3103.69 (2450.72, 4028.39) | 3196 (2542.96, 4065.41) | 7243.99 (5734.3, 9291.36) | 3210.81 (2555.89, 4082.99) | 0.46 (0.26, 0.65) |
| **Venezuela (Bolivarian Republic of)** | 185812.71 (146119.52, 241388.81) | 1357.06 (1070.75, 1739.67) | 412978.42 (322535.4, 532044.41) | 1357.61 (1071.07, 1740.34) | 0.04 (0, 0.08) |
| **Viet Nam** | 1843562.54 (1455944.22, 2324503.94) | 3830.57 (3032.03, 4841.67) | 4100702.91 (3176401.91, 5296651.1) | 3815.91 (3022.06, 4820.57) | -0.38 (-0.63, -0.14) |
| **Yemen** | 208346.14 (163308.98, 269637.63) | 2871.32 (2253.85, 3633.46) | 597237.98 (464124.77, 777152.79) | 2874.21 (2251.5, 3638.23) | 0.1 (-0.35, 0.48) |
| **Zambia** | 51689.86 (40344.65, 67163.11) | 1146.03 (904.59, 1466.43) | 133078.17 (104177.37, 174738.12) | 1146.85 (905.06, 1465.02) | 0.07 (-0.2, 0.39) |
| **Zimbabwe** | 69978 (54714.16, 90460.14) | 1148.44 (906.06, 1467.46) | 121854.02 (95536.43, 157680.68) | 1157.04 (913.52, 1476.55) | 0.75 (0.32, 1.3) |
| **Incidence** | | | | | |
| **Afghanistan** | 49015.57 (38420.42, 62947.7) | 609.41 (479.51, 789.6) | 148128.69 (110847.69, 194129.37) | 600.7 (472.24, 770.29) | -1.43 (-2.19, -0.72) |
| **Albania** | 9118.06 (7116.02, 11695.83) | 325.48 (255.3, 411.16) | 10752.53 (8485.22, 13387.44) | 326.04 (255.48, 412) | 0.17 (-0.09, 0.47) |
| **Algeria** | 105161.45 (82263.61, 135460.52) | 602.8 (473.57, 772.99) | 255805.88 (197820.04, 335480.42) | 601.88 (473.3, 773.77) | -0.15 (-0.31, 0) |
| **American Samoa** | 250.06 (194.49, 324.62) | 674.67 (533.97, 853.38) | 356.05 (279.48, 451.74) | 677.03 (536.23, 855.94) | 0.35 (0.17, 0.59) |
| **Andorra** | 358.79 (280.17, 463.95) | 591.72 (464.66, 749.6) | 611.77 (478.67, 771.81) | 594.48 (466.99, 754.22) | 0.47 (0.29, 0.69) |
| **Angola** | 18268.77 (13913.6, 23634.17) | 267.37 (208.4, 339.39) | 53933.98 (41156.07, 69791.5) | 269.03 (209.52, 341.35) | 0.62 (0.45, 0.84) |
| **Antigua and Barbuda** | 171.85 (135.03, 218.02) | 314.09 (245.03, 398.07) | 325.84 (252.9, 415.97) | 313.75 (244.63, 397.96) | -0.11 (-0.21, -0.03) |
| **Argentina** | 127446.52 (99754.13, 160837.44) | 396.75 (310.24, 501.29) | 194545.8 (152800.64, 244916.91) | 396.82 (310.34, 501.4) | 0.02 (-0.03, 0.05) |
| **Armenia** | 10391.43 (8077.79, 13223.07) | 326.74 (256.29, 413.01) | 11750.43 (9249.13, 14830.12) | 326.75 (256.25, 412.87) | 0 (-0.11, 0.09) |
| **Australia** | 45182.66 (35893.6, 56471.61) | 246.1 (194.93, 307.43) | 74124.34 (58770.49, 92219.83) | 246.19 (194.93, 307.68) | 0.04 (-0.1, 0.14) |
| **Austria** | 51879.27 (40975.37, 65244.48) | 596.48 (468.11, 755.65) | 63197.65 (49770.89, 78720.42) | 595.67 (467.56, 755.21) | -0.14 (-0.33, 0.01) |
| **Azerbaijan** | 20390.18 (15845.41, 26029.56) | 326.95 (256.48, 413.35) | 36880.34 (28480.81, 47778.3) | 326.33 (255.9, 412.46) | -0.19 (-0.36, -0.05) |
| **Bahamas** | 691.62 (536.36, 888.12) | 314 (244.88, 397.67) | 1337.06 (1033.69, 1714.13) | 313.9 (244.79, 397.72) | -0.03 (-0.08, 0.01) |
| **Bahrain** | 2482.71 (1870.92, 3319.2) | 577.41 (453.98, 736.6) | 10704.83 (7958.59, 14829.37) | 571.97 (449.82, 731.9) | -0.94 (-1.52, -0.44) |
| **Bangladesh** | 239620.97 (186271.76, 307345.18) | 324.83 (254.54, 411.08) | 502952.35 (394601.27, 639970.48) | 326.02 (255.38, 412.15) | 0.37 (0.13, 0.65) |
| **Barbados** | 809.29 (635.25, 1018.85) | 314.19 (245.04, 397.89) | 1181.59 (928.77, 1490.45) | 313.85 (244.72, 397.71) | -0.11 (-0.21, -0.03) |
| **Belarus** | 38177.55 (30060.43, 48149.17) | 327.1 (256.75, 413.69) | 40072.92 (31537.97, 50367.98) | 326.85 (256.46, 413.1) | -0.08 (-0.17, 0.01) |
| **Belgium** | 59695.62 (47497.41, 74229.16) | 528.59 (412.67, 668.1) | 70871.61 (55641.39, 87026.63) | 528.75 (412.85, 668.56) | 0.03 (-0.06, 0.1) |
| **Belize** | 395.09 (308.42, 505.41) | 312.67 (243.81, 396.19) | 1169.15 (898.82, 1500.01) | 313.17 (244.07, 397.44) | 0.16 (0.03, 0.32) |
| **Benin** | 8310.17 (6421.92, 10654.75) | 277.78 (216.48, 354.48) | 23133.29 (17648.99, 29795.1) | 276.92 (216.03, 353.13) | -0.31 (-0.42, -0.16) |
| **Bermuda** | 211.94 (164, 271.53) | 313.84 (244.69, 397.37) | 275.53 (215.69, 349.07) | 313.49 (244.42, 397) | -0.11 (-0.19, -0.04) |
| **Bhutan** | 1372.49 (1065.43, 1770.87) | 325.3 (255.05, 410.77) | 2381.88 (1863.84, 3059.29) | 325.07 (254.9, 410.5) | -0.07 (-0.2, 0.03) |
| **Bolivia (Plurinational State of)** | 14558.36 (11294.97, 18777.79) | 313.67 (244.56, 397.54) | 33782.01 (26245.29, 43150.29) | 313.32 (244.17, 397.03) | -0.11 (-0.16, -0.05) |
| **Bosnia and Herzegovina** | 15357.39 (11949.05, 19597.36) | 326.23 (255.93, 412.35) | 14233.01 (11259.58, 17774.83) | 326.17 (255.8, 412.1) | -0.02 (-0.15, 0.11) |
| **Botswana** | 2379.95 (1825.72, 3041.99) | 269.24 (209.66, 341.56) | 5776.41 (4413.14, 7548.2) | 268.68 (209.41, 341.09) | -0.21 (-0.38, -0.05) |
| **Brazil** | 624300.56 (483790.18, 809076.48) | 506.19 (396.8, 645.79) | 1225839.99 (958335.33, 1571603.86) | 503.83 (395.14, 641.96) | -0.47 (-0.7, -0.26) |
| **Brunei Darussalam** | 724.51 (552.91, 954.78) | 353.82 (277.52, 454.25) | 1668.31 (1280.62, 2205.86) | 355.81 (278.81, 457.29) | 0.56 (0.37, 0.75) |
| **Bulgaria** | 33903.21 (26779.8, 42789.92) | 326.18 (255.76, 412.14) | 30913.96 (24496.63, 38297.95) | 325.98 (255.7, 411.87) | -0.06 (-0.15, 0.03) |
| **Burkina Faso** | 16982.67 (13038.61, 21458.23) | 278.28 (216.76, 355.03) | 42120.98 (32081.12, 54278.04) | 278.03 (216.88, 354.61) | -0.09 (-0.24, 0.07) |
| **Burundi** | 9697.05 (7449.58, 12414.41) | 269.06 (209.48, 341.49) | 21426.11 (16394.83, 27613.59) | 266.91 (208.08, 338.81) | -0.8 (-1.21, -0.51) |
| **Cabo Verde** | 668.86 (526.91, 831.64) | 281.03 (218.68, 358.29) | 1479.67 (1137.74, 1904.65) | 275.71 (214.84, 351.39) | -1.89 (-2.5, -1.27) |
| **Cambodia** | 54308.72 (42229.51, 69975.11) | 780.67 (615.8, 994.91) | 120355.28 (93353.05, 155838.92) | 777.96 (612.34, 991.85) | -0.35 (-0.62, -0.12) |
| **Cameroon** | 18966.34 (14559.11, 24146.99) | 276.52 (215.7, 352.75) | 57367.11 (43504.69, 74286.85) | 276.09 (215.29, 352.15) | -0.16 (-0.23, -0.08) |
| **Canada** | 163736.16 (128834.83, 207806.14) | 533.86 (419.09, 675.8) | 243895.4 (190987.91, 305755.38) | 534.31 (419.47, 676.66) | 0.08 (0, 0.16) |
| **Central African Republic** | 5017.99 (3830.5, 6444.21) | 268.79 (209.39, 341.06) | 10124.07 (7751.24, 13091.34) | 268.43 (209.21, 340.65) | -0.13 (-0.33, 0.03) |
| **Chad** | 10698.62 (8268.35, 13599.67) | 277.83 (216.45, 354.5) | 26755.16 (20448.64, 34054.44) | 275.58 (214.95, 351.51) | -0.81 (-1.12, -0.54) |
| **Chile** | 48543.98 (37671.71, 62100.11) | 397.25 (310.71, 502.11) | 83784.68 (65783.63, 104946.94) | 396.51 (310.13, 500.97) | -0.19 (-0.24, -0.13) |
| **China** | 8930797.12 (7008104.84, 11430250.8) | 800.68 (634.43, 1016.63) | 14887967.2 (11666334.23, 19183944.68) | 809.91 (641.89, 1028.61) | 1.15 (-2.46, 5.09) |
| **Colombia** | 81379.98 (63057.64, 104925.13) | 313.39 (244.27, 397.3) | 161551.9 (125758.96, 203970.18) | 313.86 (244.76, 397.72) | 0.15 (0.03, 0.25) |
| **Comoros** | 832.94 (640.36, 1055.84) | 268.34 (208.88, 340.5) | 1688.81 (1298, 2170.94) | 268 (208.86, 340.22) | -0.13 (-0.32, 0.05) |
| **Congo** | 4375.62 (3361.34, 5564.39) | 268.95 (209.51, 341.39) | 11483.74 (8723.03, 14965.62) | 267.68 (208.6, 339.76) | -0.47 (-0.76, -0.28) |
| **Cook Islands** | 107.87 (84.93, 137.55) | 674.63 (533.85, 853.24) | 137.43 (107.37, 172.45) | 678.9 (537.61, 859) | 0.63 (0.37, 0.94) |
| **Costa Rica** | 7568.09 (5891.43, 9770.18) | 313.29 (244.14, 397.12) | 16250.87 (12657.16, 20604.75) | 313.9 (244.79, 397.91) | 0.2 (0.1, 0.3) |
| **Croatia** | 18828.06 (14799.42, 23867.77) | 326.43 (256.14, 412.45) | 18542.63 (14782.55, 22953.18) | 326.08 (255.69, 411.91) | -0.11 (-0.24, 0.03) |
| **Cuba** | 34060.23 (26542.2, 43194.66) | 313.17 (243.99, 397.06) | 45819.49 (35787.02, 58377.44) | 313.22 (244.07, 396.85) | 0.01 (-0.06, 0.1) |
| **Cyprus** | 4799.23 (3777.08, 6079.29) | 595.81 (467.54, 755.38) | 9199.71 (7237.33, 11691.43) | 597.53 (468.75, 757.78) | 0.29 (0.12, 0.49) |
| **Czechia** | 38934.54 (30771.28, 48765.35) | 326.44 (256.09, 412.42) | 46379.28 (36751.45, 58003.23) | 325.87 (255.55, 411.56) | -0.17 (-0.29, -0.06) |
| **Côte d'Ivoire** | 21321.1 (16108.84, 27572.86) | 273.85 (213.6, 348.85) | 52981.9 (40046.59, 69127.3) | 273.89 (213.48, 349.16) | 0.01 (-0.09, 0.12) |
| **Democratic People's Republic of Korea** | 131734.83 (101758.05, 167625.11) | 665.41 (522.69, 836.24) | 205668.55 (159966.19, 261159.57) | 660.06 (518.38, 829.83) | -0.8 (-1.07, -0.58) |
| **Democratic Republic of the Congo** | 67277.3 (51632.27, 85729.2) | 268.97 (209.37, 341.45) | 163839.82 (125190.87, 211713.15) | 268.05 (209.03, 340.22) | -0.34 (-0.6, -0.1) |
| **Denmark** | 40597.25 (32378.29, 50793.72) | 696.29 (547.19, 881.35) | 46303.36 (36603.14, 57393.37) | 696.26 (547.27, 881.31) | 0 (-0.1, 0.06) |
| **Djibouti** | 819.34 (623.68, 1068.13) | 266.49 (208.01, 338.4) | 2756.6 (2100.08, 3618.3) | 266.41 (207.64, 338.33) | -0.03 (-0.27, 0.23) |
| **Dominica** | 200.78 (158.87, 251.01) | 313.78 (244.62, 397.18) | 243.13 (190.54, 306.72) | 312.72 (243.75, 396.29) | -0.34 (-0.72, 0.03) |
| **Dominican Republic** | 16948.04 (13177.7, 21871.26) | 313.44 (244.27, 397.55) | 33245.12 (25823.88, 42449.39) | 313.09 (243.96, 396.66) | -0.11 (-0.24, 0.01) |
| **Ecuador** | 23768.81 (18447.38, 30583.87) | 313.25 (244.12, 397.12) | 53034.75 (41251.19, 67401.32) | 313.43 (244.29, 397.35) | 0.06 (0.01, 0.1) |
| **Egypt** | 254662.58 (197285.42, 331314.62) | 600.95 (472.52, 772.08) | 529837.47 (410943.12, 693416.85) | 598.07 (470.77, 768.39) | -0.48 (-0.69, -0.32) |
| **El Salvador** | 12188.02 (9491.68, 15647.98) | 313.95 (244.84, 397.99) | 19157.82 (14939.81, 24252.64) | 315.11 (245.82, 400.1) | 0.37 (0.16, 0.57) |
| **Equatorial Guinea** | 764.84 (587.9, 970.15) | 269.97 (210.11, 342.95) | 2622.62 (1992.84, 3422.96) | 269.04 (209.8, 341.72) | -0.35 (-0.74, 0.02) |
| **Eritrea** | 5124.54 (3885.85, 6622.46) | 269.19 (209.79, 341.71) | 13259.38 (10107.16, 17256.21) | 268.39 (209.33, 340.77) | -0.3 (-0.45, -0.19) |
| **Estonia** | 5854.76 (4605.63, 7352.98) | 327.16 (256.81, 413.86) | 5601.73 (4453.82, 6915.85) | 326.35 (256.09, 412.21) | -0.25 (-0.42, -0.09) |
| **Eswatini** | 1358.41 (1034.94, 1746.1) | 269.34 (209.74, 342.18) | 2448.3 (1875.45, 3180.27) | 269.31 (209.9, 341.89) | -0.01 (-0.27, 0.29) |
| **Ethiopia** | 104939.95 (80870.56, 136028.45) | 321.28 (253.95, 408.71) | 235214.25 (181139.43, 306887.42) | 321.21 (253.86, 408.65) | -0.02 (-0.14, 0.08) |
| **Fiji** | 4134.16 (3204.9, 5380.9) | 676.45 (535.72, 854.97) | 6109.73 (4807.79, 7835.6) | 675.73 (535.11, 853.67) | -0.11 (-0.19, -0.01) |
| **Finland** | 38143.71 (30209.33, 48385.52) | 670.7 (530.65, 856.04) | 43526.73 (34953.86, 53343.66) | 671.89 (530.58, 849.84) | 0.18 (-3.88, 5.25) |
| **France** | 323530.33 (258269.02, 401185.98) | 507.18 (398.25, 634.25) | 391812.3 (310957.96, 480563.89) | 507.58 (398.72, 635.32) | 0.08 (-0.02, 0.17) |
| **Gabon** | 1922.2 (1492.93, 2421.68) | 268.05 (208.96, 340.23) | 4039.6 (3077.54, 5205.35) | 268.44 (209.06, 340.56) | 0.15 (-0.19, 0.46) |
| **Gambia** | 1706.98 (1308.38, 2200.51) | 273.81 (213.4, 348.72) | 4375.86 (3350.4, 5656.54) | 276.06 (215.33, 351.99) | 0.82 (0.57, 1.11) |
| **Georgia** | 19187.19 (15053.98, 24253.52) | 327.1 (256.66, 413.66) | 14687.65 (11595.15, 18302.12) | 326.56 (256.18, 412.61) | -0.17 (-0.3, -0.05) |
| **Germany** | 544896.4 (429703.31, 686400.93) | 595.98 (467.9, 754.96) | 607187.81 (476304.6, 754636.75) | 594.75 (466.76, 753.91) | -0.21 (-0.43, -0.04) |
| **Ghana** | 28082.47 (21499.58, 36145.81) | 276.26 (215.43, 352.45) | 70497.85 (53803.74, 91307.72) | 277.85 (216.64, 354.32) | 0.57 (0.41, 0.77) |
| **Greece** | 72821.77 (58052.96, 90218.09) | 630.58 (497.19, 796.24) | 79266.28 (62884.94, 97744.47) | 630.74 (497.39, 796.52) | 0.03 (-0.03, 0.07) |
| **Greenland** | 290.78 (220.36, 376.77) | 526.69 (414.04, 664.34) | 342.35 (264.64, 447.35) | 531.52 (417.85, 671.64) | 0.92 (0.54, 1.31) |
| **Grenada** | 215.23 (170.07, 270.54) | 313.91 (244.75, 397.41) | 355.08 (276.07, 452.07) | 312.89 (244.03, 396.51) | -0.32 (-0.55, -0.13) |
| **Guam** | 828.05 (645.6, 1080.04) | 673.09 (532.79, 850.28) | 1201.88 (941.97, 1509.31) | 675.02 (534.44, 853.41) | 0.29 (0.11, 0.47) |
| **Guatemala** | 16937.97 (13061.89, 21607.82) | 313.35 (244.17, 397.37) | 46944.91 (36537.69, 60645.57) | 314.48 (245.36, 399.05) | 0.36 (0.15, 0.55) |
| **Guinea** | 11918.09 (9286.02, 15169.36) | 276.9 (215.94, 353.35) | 23585.46 (18152.07, 30313.93) | 277.2 (216, 353.68) | 0.11 (0.02, 0.2) |
| **Guinea-Bissau** | 1791.09 (1376.69, 2300.88) | 277.94 (216.51, 354.6) | 3684.29 (2794.49, 4782.9) | 277.65 (216.55, 354.07) | -0.1 (-0.22, 0.04) |
| **Guyana** | 1840.46 (1426.55, 2390.93) | 313.36 (244.27, 397.18) | 2348.3 (1816.91, 3011.09) | 313.59 (244.5, 397.41) | 0.07 (0.03, 0.12) |
| **Haiti** | 14577.55 (11288.61, 18734.64) | 313.58 (244.54, 397.58) | 32963.63 (25510.4, 42985.33) | 314.02 (244.89, 398.21) | 0.14 (0.02, 0.26) |
| **Honduras** | 9637.37 (7463.93, 12368.84) | 313.39 (244.23, 397.36) | 25813.77 (20031.26, 33223.53) | 314.03 (244.87, 398.2) | 0.2 (0.1, 0.32) |
| **Hungary** | 40255.14 (31818.25, 50743.07) | 326.59 (256.2, 412.58) | 42595.85 (33704, 52960.29) | 326.33 (255.99, 412.37) | -0.08 (-0.15, -0.02) |
| **Iceland** | 1562.06 (1233.32, 1983.22) | 595.45 (467.47, 754.44) | 2301.1 (1823.37, 2879.94) | 594.96 (466.87, 753.91) | -0.08 (-0.16, -0.02) |
| **India** | 2633194.66 (2039451.45, 3398487.45) | 391.37 (305.81, 492.84) | 5332091.96 (4164005.03, 6822808.19) | 392.03 (306.34, 493.29) | 0.17 (0.07, 0.26) |
| **Indonesia** | 1432389.76 (1108988.13, 1844182.91) | 965.78 (764.37, 1220.3) | 2649930.25 (2051113.64, 3411548.99) | 965 (763.65, 1218.88) | -0.08 (-0.14, -0.02) |
| **Iran (Islamic Republic of)** | 367706.63 (286731.31, 474622.04) | 929 (729.55, 1187.68) | 865873.46 (668905.91, 1146824.17) | 934.14 (732.88, 1189.94) | 0.55 (0.26, 0.9) |
| **Iraq** | 69077.92 (53721.59, 89541.96) | 600.43 (472.14, 770.65) | 215073.49 (165120.74, 281624.14) | 600.2 (471.93, 770.61) | -0.04 (-0.09, 0.01) |
| **Ireland** | 21886.4 (17220.1, 27455.69) | 596.07 (468.13, 755.77) | 33136.6 (26113.1, 41573.21) | 597.11 (468.75, 757.6) | 0.17 (0.05, 0.28) |
| **Israel** | 28810.68 (22649.35, 36428.84) | 597.45 (468.79, 757.76) | 56566.31 (44742.7, 71052.44) | 596.38 (468.01, 756.1) | -0.18 (-0.26, -0.1) |
| **Italy** | 507289.07 (404935.22, 629727.65) | 774.85 (612.82, 975.97) | 582882.42 (460736.14, 720926.3) | 773.46 (611.97, 973.96) | -0.18 (-0.26, -0.11) |
| **Jamaica** | 5995.08 (4707.46, 7620.83) | 313.56 (244.48, 397.33) | 9418.51 (7353.76, 11989.02) | 313.39 (244.37, 397.47) | -0.05 (-0.15, 0.04) |
| **Japan** | 680099.08 (534655, 863460.11) | 443.92 (348.96, 564.14) | 798968 (631030.89, 989719.65) | 442.76 (348.05, 562.75) | -0.26 (-0.4, -0.17) |
| **Jordan** | 14257.01 (10959, 18523.72) | 597.71 (470.31, 768.17) | 61401.83 (47291.45, 80947.31) | 592.94 (466.91, 759.5) | -0.8 (-1.13, -0.56) |
| **Kazakhstan** | 48931.47 (37991.28, 62224.17) | 327.07 (256.7, 413.7) | 62545.19 (48519.04, 79991.45) | 326.86 (256.43, 413) | -0.07 (-0.17, 0.04) |
| **Kenya** | 45253.22 (34868.7, 58742.67) | 321.64 (254.21, 409.02) | 122029.75 (93863.76, 158038.5) | 321.78 (254.47, 409.09) | 0.05 (-0.04, 0.15) |
| **Kiribati** | 384.76 (301.32, 497.61) | 678.17 (537.35, 856.86) | 689.38 (536.38, 890.73) | 679.28 (538.5, 857.69) | 0.16 (0.06, 0.28) |
| **Kuwait** | 7143.71 (5360.08, 9572.77) | 475.32 (370.06, 603.32) | 25771.15 (19075.11, 34888.37) | 488.69 (379.9, 623.23) | 2.81 (2.12, 3.46) |
| **Kyrgyzstan** | 11733.89 (9188.11, 14946.18) | 326.82 (256.46, 413.23) | 19483.29 (15052.16, 25060.48) | 326.57 (256.13, 412.61) | -0.08 (-0.21, 0.03) |
| **Lao People's Democratic Republic** | 22980.96 (17871.06, 29588.73) | 778.06 (612.38, 992) | 49802.93 (38382.92, 64746.55) | 777.21 (611.49, 991.07) | -0.11 (-0.22, -0.02) |
| **Latvia** | 10038.77 (7908.13, 12561.61) | 327.22 (256.85, 413.96) | 8304.18 (6590.48, 10179.69) | 326.69 (256.31, 412.82) | -0.16 (-0.29, -0.03) |
| **Lebanon** | 16291.28 (12728.05, 21008.86) | 605.74 (476.1, 781.97) | 32864.97 (25812.61, 42571.92) | 608.36 (477.48, 782.11) | 0.43 (0.12, 0.8) |
| **Lesotho** | 3521.81 (2721.49, 4446.68) | 268.51 (209.26, 340.79) | 4871.39 (3753.31, 6264.82) | 268.7 (209.57, 341.15) | 0.07 (-0.15, 0.29) |
| **Liberia** | 3847.15 (2989.37, 4898.13) | 275.02 (214.48, 350.72) | 10045.69 (7653.92, 13140.97) | 274.78 (214.27, 350.43) | -0.09 (-0.21, 0.05) |
| **Libya** | 16644.37 (12909.58, 21548) | 589.97 (464.54, 754.42) | 44327 (33932.73, 59597.62) | 598.51 (470.84, 767.39) | 1.45 (1.11, 1.84) |
| **Lithuania** | 13328.82 (10477.9, 16828.53) | 327.05 (256.67, 413.55) | 12069.02 (9532.02, 14760.72) | 326.72 (256.32, 412.9) | -0.1 (-0.18, -0.02) |
| **Luxembourg** | 2583.57 (2030.21, 3266.74) | 595.6 (467.64, 754.45) | 4330.87 (3421.37, 5452.14) | 595.18 (467.46, 754.78) | -0.07 (-0.29, 0.07) |
| **Madagascar** | 21073.27 (16196.63, 27001.57) | 267.89 (208.47, 339.92) | 51968.98 (39727.04, 67290.79) | 268.02 (208.73, 340.09) | 0.05 (-0.02, 0.15) |
| **Malawi** | 16676.92 (12775.69, 21437.5) | 268.39 (209.02, 340.57) | 33883.99 (25884.55, 43517.89) | 268.58 (209.25, 340.82) | 0.07 (-0.02, 0.18) |
| **Malaysia** | 82893.97 (66149.08, 102794.19) | 595.11 (479.85, 729.08) | 210158.2 (165068.05, 270526.73) | 653.17 (513.72, 827) | 9.76 (2.9, 17.97) |
| **Maldives** | 1106.81 (857.22, 1424.06) | 777.11 (611.39, 991.11) | 4151.53 (3152.41, 5474.69) | 770.84 (606.65, 983.67) | -0.81 (-1.49, -0.26) |
| **Mali** | 16050.24 (12313.47, 20473.92) | 276.54 (215.72, 352.85) | 38394.63 (29482.03, 48684.06) | 275.82 (215.19, 351.9) | -0.26 (-0.37, -0.18) |
| **Malta** | 2398.79 (1890.52, 3053.23) | 596.48 (467.92, 755.89) | 3135.33 (2500.01, 3895.33) | 594.81 (466.85, 753.78) | -0.28 (-0.4, -0.16) |
| **Marshall Islands** | 195.72 (152.95, 253.5) | 674.46 (533.93, 852.74) | 350.04 (273.69, 454.02) | 676.09 (535.42, 854.06) | 0.24 (0.06, 0.41) |
| **Mauritania** | 3879.35 (2999.98, 4958.66) | 276.56 (215.72, 352.71) | 8281.23 (6361.71, 10578.4) | 276.73 (215.9, 353.01) | 0.06 (-0.04, 0.16) |
| **Mauritius** | 7836.68 (6098.13, 10175.66) | 777.11 (611.29, 990.7) | 12230.01 (9610.5, 15538.73) | 776.94 (611.18, 990.59) | -0.02 (-0.07, 0.02) |
| **Mexico** | 238360.68 (185049.7, 307643.29) | 376.96 (296.66, 479.95) | 485590.38 (379047.28, 626707.52) | 377.28 (296.9, 480.24) | 0.08 (0.03, 0.14) |
| **Micronesia (Federated States of)** | 485.08 (382.12, 625.13) | 674.99 (534.38, 853.14) | 634.1 (491.45, 812.74) | 676.17 (535.51, 853.9) | 0.17 (0.05, 0.3) |
| **Monaco** | 223.99 (179.12, 279.09) | 596.91 (468.5, 756.82) | 273.92 (215.4, 340.77) | 597.87 (469.41, 758.44) | 0.16 (-0.04, 0.36) |
| **Mongolia** | 4950.74 (3861.93, 6343.08) | 326.16 (255.77, 411.92) | 10887.49 (8337.22, 14240.41) | 326.58 (256.19, 412.63) | 0.13 (0.02, 0.26) |
| **Montenegro** | 2106.61 (1648.02, 2674.65) | 326.24 (255.84, 412.12) | 2518.8 (1984.98, 3154.11) | 326.12 (255.69, 412.13) | -0.04 (-0.12, 0.04) |
| **Morocco** | 116161.8 (90342.16, 150253.77) | 603.95 (474.6, 776.14) | 223898.34 (174038.94, 290470.79) | 602.84 (473.83, 774.96) | -0.18 (-0.26, -0.06) |
| **Mozambique** | 24082.93 (18432.03, 30903.44) | 269.02 (209.37, 341.59) | 51140.33 (38994.95, 65744.08) | 269.17 (209.64, 341.44) | 0.05 (-0.09, 0.2) |
| **Myanmar** | 250123.74 (194405.56, 322859.48) | 777.7 (612.1, 991.48) | 429048.17 (334675.22, 554192.01) | 778.63 (613.2, 992.54) | 0.12 (-0.01, 0.23) |
| **Namibia** | 2662.1 (2052.83, 3375.45) | 268.53 (209.11, 340.8) | 5372.41 (4106.87, 6923.02) | 269.08 (209.67, 341.56) | 0.21 (0.1, 0.34) |
| **Nauru** | 49.9 (38.5, 65.24) | 674.33 (533.81, 852.72) | 57.2 (43.9, 75.02) | 678.63 (538.37, 855.95) | 0.64 (0.29, 1.02) |
| **Nepal** | 45630.59 (35353, 58470.22) | 325.81 (255.27, 411.95) | 89641.03 (70148.62, 114100.47) | 327.08 (256.46, 413.23) | 0.39 (0.13, 0.67) |
| **Netherlands** | 92506.17 (74049.9, 117325.86) | 556.17 (443.11, 706.05) | 111739.04 (88262.22, 138780.01) | 556.51 (443.18, 706.44) | 0.06 (-0.09, 0.2) |
| **New Zealand** | 10096.23 (7842.47, 12947.88) | 277.24 (213.97, 355.98) | 11775.24 (9507.56, 14548.15) | 212.7 (170.55, 262.98) | -23.28 (-29.15, -17.15) |
| **Nicaragua** | 7728.13 (5992.7, 9987.65) | 313.77 (244.77, 397.89) | 18575.47 (14300.2, 23910.06) | 313.92 (244.8, 397.73) | 0.05 (-0.07, 0.17) |
| **Niger** | 13431.28 (10217.79, 17245.32) | 275.15 (214.65, 350.7) | 37072.78 (28431.77, 47067.52) | 276.95 (216.12, 353.24) | 0.66 (0.42, 0.95) |
| **Nigeria** | 210396.81 (163444.52, 270069.29) | 332.61 (262.87, 420.93) | 513547.39 (394156.55, 667072.67) | 348.46 (274.27, 443.56) | 4.76 (1.35, 8.19) |
| **Niue** | 13.87 (11.01, 17.37) | 676.82 (536.09, 854.91) | 12.53 (9.86, 15.75) | 676.45 (535.75, 854.81) | -0.05 (-0.21, 0.07) |
| **North Macedonia** | 6689.67 (5231.7, 8501.99) | 325.93 (255.54, 411.77) | 8885.4 (6998.41, 11224.7) | 325.61 (255.28, 411.21) | -0.1 (-0.2, -0.02) |
| **Northern Mariana Islands** | 284.41 (215.29, 384.65) | 668.13 (528.61, 846.77) | 345.09 (263.86, 458.5) | 674.49 (533.71, 853.18) | 0.95 (0.35, 1.65) |
| **Norway** | 31870.38 (25488.44, 39736.59) | 658.78 (523.61, 833.31) | 36125.41 (28450.89, 44553.37) | 576.71 (453.67, 723.4) | -12.46 (-15.79, -8.3) |
| **Oman** | 7968.15 (6068.83, 10508.67) | 573.22 (450.89, 729.41) | 26383.76 (19791.2, 35700.93) | 567.83 (446.09, 724.06) | -0.94 (-1.24, -0.59) |
| **Pakistan** | 304142.03 (236589.58, 387014) | 391.01 (305.49, 492.41) | 670778.59 (517978.4, 865745.14) | 391.86 (306.12, 493.25) | 0.22 (0.12, 0.35) |
| **Palau** | 93.22 (73.13, 120.53) | 675.67 (535.1, 853.84) | 150.02 (116.62, 194.12) | 671.18 (531.42, 847.71) | -0.66 (-1.02, -0.29) |
| **Palestine** | 7576.56 (5859.68, 9720.59) | 608.21 (477.17, 779.71) | 23148.01 (17783.42, 30171.69) | 601.22 (472.72, 772.97) | -1.15 (-1.7, -0.72) |
| **Panama** | 6141.28 (4779.93, 7880.62) | 312.85 (243.85, 396.61) | 13169.56 (10258.44, 16758.19) | 313.11 (244.01, 396.93) | 0.08 (0.03, 0.14) |
| **Papua New Guinea** | 20374.42 (15810.18, 26361.79) | 674.9 (534.26, 853.3) | 54019.5 (41939.16, 70364.88) | 675.49 (534.64, 854.76) | 0.09 (-0.02, 0.18) |
| **Paraguay** | 11172.97 (8634.75, 14386.23) | 373.45 (290.93, 478.31) | 24718.42 (19174.99, 32104.62) | 373.41 (290.92, 478.21) | -0.01 (-0.04, 0.02) |
| **Peru** | 51986.03 (40292.28, 66878.58) | 313.3 (244.2, 397.2) | 107956.83 (83851.01, 137686.46) | 313.44 (244.27, 397.42) | 0.04 (-0.02, 0.09) |
| **Philippines** | 543558.32 (421125.46, 700309.92) | 1157.2 (917.95, 1462.4) | 1183462.67 (923660, 1514634.33) | 1156.25 (917.09, 1461.47) | -0.08 (-0.17, -0.01) |
| **Poland** | 163663.37 (127985.35, 205832.44) | 393.23 (307.31, 494.83) | 199317.91 (157802.9, 250508.87) | 392.75 (306.94, 494.38) | -0.12 (-0.19, -0.06) |
| **Portugal** | 66215.33 (52296.07, 82667.49) | 598.05 (469.13, 758.21) | 77999.2 (61946.61, 97138.5) | 597.91 (469.09, 758.36) | -0.02 (-0.08, 0.02) |
| **Puerto Rico** | 11340.18 (8846.55, 14386.89) | 314.03 (244.93, 398.14) | 14277.88 (11323.37, 17770.22) | 313.99 (244.91, 397.87) | -0.01 (-0.07, 0.05) |
| **Qatar** | 2321.9 (1730.75, 3203.39) | 555.04 (436.07, 706.11) | 18094.95 (13388.28, 24750.13) | 547.01 (429.56, 697.75) | -1.45 (-2.01, -0.97) |
| **Republic of Korea** | 151475.5 (117413.74, 198529.73) | 358.31 (280.32, 460.26) | 258217.02 (201520.01, 332444.35) | 356.64 (279.43, 458.59) | -0.47 (-0.66, -0.29) |
| **Republic of Moldova** | 14879.95 (11652.36, 18889.14) | 327.07 (256.6, 413.37) | 15539.78 (12250.32, 19596.56) | 326.55 (256.21, 412.51) | -0.16 (-0.28, -0.05) |
| **Romania** | 83639.41 (66053.06, 105144.85) | 326.24 (255.85, 412.37) | 83135.61 (65682.92, 103276.62) | 326.05 (255.76, 411.95) | -0.06 (-0.12, 0) |
| **Russian Federation** | 665958.39 (519104.35, 844013.3) | 394.24 (308.15, 496.01) | 733935.86 (574953.77, 923934.39) | 393.79 (307.75, 495.45) | -0.11 (-0.25, -0.01) |
| **Rwanda** | 12366.66 (9539.72, 15819.16) | 269.24 (209.65, 341.77) | 26224.92 (20128.5, 34000.42) | 269.34 (209.91, 341.91) | 0.04 (-0.04, 0.14) |
| **Saint Kitts and Nevis** | 108.99 (85.8, 136.83) | 313.77 (244.76, 397.4) | 219.44 (168.74, 282.42) | 313.01 (244.1, 396.66) | -0.24 (-0.4, -0.09) |
| **Saint Lucia** | 328.68 (256.96, 420.37) | 313.97 (244.95, 397.74) | 650.79 (507.84, 829.59) | 313.29 (244.25, 397.12) | -0.22 (-0.36, -0.09) |
| **Saint Vincent and the Grenadines** | 261.98 (204.92, 333.58) | 313.57 (244.47, 397.03) | 398.09 (310.33, 504.34) | 312.72 (243.78, 396.67) | -0.27 (-0.52, -0.07) |
| **Samoa** | 807.61 (637.37, 1027.72) | 675.63 (534.94, 854.1) | 1219.39 (952.76, 1550.63) | 675.14 (534.53, 853.43) | -0.07 (-0.2, 0.08) |
| **San Marino** | 153.58 (121.21, 191.52) | 595.36 (467.31, 753.93) | 232.36 (184.22, 289.07) | 599.7 (470.84, 760.8) | 0.73 (0.39, 1.18) |
| **Sao Tome and Principe** | 220.96 (172.25, 276.55) | 277.08 (216.22, 353.38) | 457.65 (349.92, 590.93) | 275.65 (214.95, 351.52) | -0.52 (-0.77, -0.31) |
| **Saudi Arabia** | 65755.14 (50326.55, 86245.44) | 578.7 (454.97, 738.43) | 231329.14 (172638.58, 310834.44) | 580.19 (455.89, 741.83) | 0.26 (-0.06, 0.47) |
| **Senegal** | 13451.45 (10370.32, 17090.95) | 276.58 (215.75, 352.84) | 30885.61 (23702.72, 39578.2) | 276.83 (216.02, 353.16) | 0.09 (-0.02, 0.2) |
| **Serbia** | 34811.42 (27348.01, 44207.4) | 326.1 (255.71, 411.87) | 36383.01 (28958.21, 44998.28) | 325.98 (255.61, 411.8) | -0.04 (-0.08, 0) |
| **Seychelles** | 485.47 (380.43, 615.62) | 775.77 (609.83, 989.27) | 920.43 (715.75, 1183.34) | 774.53 (608.55, 987.91) | -0.16 (-0.34, 0.02) |
| **Sierra Leone** | 7175.65 (5569.55, 9188.94) | 276.05 (215.39, 352.22) | 16430.23 (12577.79, 21284.84) | 275.45 (214.84, 351.23) | -0.22 (-0.3, -0.11) |
| **Singapore** | 11277.34 (8735.04, 14814.48) | 358.6 (280.85, 461.52) | 26929.08 (20970.17, 35032.14) | 356.76 (279.75, 457.67) | -0.51 (-0.8, -0.23) |
| **Slovakia** | 18477.93 (14550.24, 23223.33) | 326.48 (256.07, 412.49) | 23246.35 (18398.39, 29275.82) | 326.11 (255.78, 411.99) | -0.11 (-0.22, -0.03) |
| **Slovenia** | 7361.01 (5786.73, 9275.32) | 326.44 (256.22, 412.4) | 9133.12 (7242.35, 11344.51) | 325.66 (255.37, 411.14) | -0.24 (-0.44, -0.02) |
| **Solomon Islands** | 1508.59 (1178.66, 1944.43) | 674.76 (533.92, 853.98) | 3476.63 (2703.51, 4510.87) | 676.77 (535.97, 855.94) | 0.3 (0.13, 0.5) |
| **Somalia** | 12552.28 (9597.12, 16337.57) | 268.21 (208.91, 340.49) | 34730.88 (26576.1, 45360.41) | 268.48 (209.38, 340.99) | 0.1 (-0.08, 0.32) |
| **South Africa** | 94581.73 (73702.58, 122191.01) | 323.08 (255.49, 410.96) | 178028.46 (138187.91, 230122.43) | 322.82 (255.27, 410.36) | -0.08 (-0.16, -0.01) |
| **South Sudan** | 10258.71 (7866.14, 13113.43) | 265.62 (207.36, 337.32) | 17108.43 (12995.76, 22045.63) | 268.04 (208.44, 340.66) | 0.91 (0.58, 1.28) |
| **Spain** | 210776.08 (167050, 261913.33) | 495.25 (390.79, 625.12) | 280068.78 (220273.07, 344305.62) | 494.66 (390.39, 624.29) | -0.12 (-0.2, -0.06) |
| **Sri Lanka** | 118418.15 (92017.69, 153602.06) | 777.48 (611.88, 991.63) | 190117.85 (149185.74, 242079.97) | 777.98 (612.52, 991.73) | 0.06 (-0.1, 0.21) |
| **Sudan** | 81806.23 (63737.42, 106064.17) | 602.73 (474.34, 776.65) | 185517.27 (142954.05, 243359.86) | 602.3 (474.01, 778.82) | -0.07 (-0.25, 0.15) |
| **Suriname** | 1015.03 (786.55, 1294.07) | 313.39 (244.31, 397.51) | 1925.52 (1490.1, 2449.8) | 313.56 (244.41, 397.21) | 0.06 (-0.06, 0.18) |
| **Sweden** | 74862.69 (59689.77, 92895.21) | 760.63 (600.75, 956.11) | 90131.85 (71389.48, 111461.89) | 757.55 (598.39, 952.07) | -0.41 (-0.81, -0.02) |
| **Switzerland** | 46678.39 (36853.77, 59086.71) | 595.49 (467.26, 754.42) | 62260.13 (49033.15, 77402.38) | 595.31 (467.42, 754.76) | -0.03 (-0.16, 0.06) |
| **Syrian Arab Republic** | 48264.24 (37481.57, 62212.65) | 600.09 (472.09, 770.97) | 87383.8 (67302, 112607.95) | 608.27 (478.87, 787.13) | 1.36 (0.96, 1.83) |
| **Taiwan (Province of China)** | 133518.28 (105144.38, 171507.81) | 667.12 (525.19, 842.98) | 196590.9 (161017.04, 238205.51) | 613.75 (506.43, 735.49) | -8 (-18.54, 3.92) |
| **Tajikistan** | 12205.4 (9508.7, 15620.68) | 326.26 (255.97, 412.42) | 26249.8 (20149.58, 34012.74) | 326.02 (255.59, 411.77) | -0.07 (-0.27, 0.1) |
| **Thailand** | 399482.67 (307803.4, 518084.16) | 777.68 (612.2, 991.41) | 703747.59 (549559.56, 901697.83) | 777.69 (612.02, 991.5) | 0 (-0.04, 0.03) |
| **Timor-Leste** | 4322.84 (3303.6, 5678.07) | 776.68 (610.8, 990.89) | 8028.07 (6290.74, 10253.65) | 776.91 (611.34, 991.08) | 0.03 (-0.06, 0.13) |
| **Togo** | 6235.25 (4744.65, 8005.35) | 277.94 (216.61, 354.54) | 16817.04 (12824.34, 21754.53) | 277.75 (216.51, 354.12) | -0.07 (-0.3, 0.17) |
| **Tokelau** | 8.94 (7.11, 11.2) | 680.51 (539.58, 859.75) | 8.98 (7.04, 11.31) | 676.31 (535.87, 854.89) | -0.62 (-1.1, -0.32) |
| **Tonga** | 484.94 (382.53, 612.41) | 678.91 (537.83, 859.25) | 600.73 (474.79, 763.06) | 678.14 (537.15, 857.89) | -0.11 (-0.24, 0.01) |
| **Trinidad and Tobago** | 3272.93 (2560.17, 4200.56) | 313.25 (244.22, 396.97) | 5228.55 (4083.85, 6648.12) | 313.05 (244.01, 396.63) | -0.06 (-0.1, -0.03) |
| **Tunisia** | 39497.77 (30749.52, 51001.44) | 602.25 (473.56, 774.23) | 79382.66 (62002.19, 102496.56) | 604.32 (474.87, 777.36) | 0.34 (0.24, 0.53) |
| **Turkey** | 290176.85 (225462.41, 375885.9) | 601.94 (472.88, 772.22) | 564531.98 (440731.71, 733058.17) | 601.48 (472.59, 771.66) | -0.08 (-0.14, -0.03) |
| **Turkmenistan** | 8822.44 (6849.86, 11322.07) | 326.71 (256.35, 413.01) | 15985.98 (12369.96, 20566.92) | 325.91 (255.65, 411.66) | -0.24 (-0.49, -0.05) |
| **Tuvalu** | 55.95 (44.62, 71.51) | 681.94 (540.54, 862.73) | 76.48 (60.25, 96.72) | 675.12 (534.85, 851.68) | -1 (-1.5, -0.53) |
| **Uganda** | 28158.48 (21506.07, 36084.58) | 268.09 (208.71, 340.19) | 69664.63 (53027.83, 89555.2) | 269.07 (209.58, 341.44) | 0.37 (0.24, 0.53) |
| **Ukraine** | 240596.56 (188040.33, 301497.82) | 394.26 (308.09, 495.95) | 228590.45 (179295.81, 285923.27) | 393.89 (307.8, 495.59) | -0.09 (-0.19, -0.02) |
| **United Arab Emirates** | 9167.19 (6806.43, 12660.26) | 556.15 (436.85, 706.83) | 70712.38 (50498.3, 101158.57) | 551.03 (432.8, 700.38) | -0.92 (-1.65, -0.37) |
| **United Kingdom** | 545712.94 (437732.88, 668991.46) | 827.15 (657.72, 1033.59) | 662700.79 (527440.56, 810120.42) | 851.18 (675.9, 1073.5) | 2.9 (-1.36, 6.98) |
| **United Republic of Tanzania** | 44709.97 (34312.35, 56907.04) | 268.53 (208.95, 340.72) | 106757.11 (81587.36, 137684.22) | 268.54 (209.02, 340.71) | 0.01 (-0.02, 0.04) |
| **United States of America** | 2367059.05 (1879674.26, 2988699.37) | 841.68 (667.47, 1068.84) | 3654042.78 (2953518.69, 4414295.5) | 957.71 (771.71, 1170.78) | 13.79 (7.68, 19.71) |
| **United States Virgin Islands** | 328.83 (250.92, 427.08) | 314.02 (244.88, 398.09) | 408.25 (319.44, 515.23) | 314.18 (245.09, 398.18) | 0.05 (0, 0.1) |
| **Uruguay** | 13127.19 (10369.11, 16381.85) | 396.9 (310.43, 501.56) | 15874.59 (12547.9, 19702.23) | 397.07 (310.56, 501.83) | 0.04 (0.02, 0.07) |
| **Uzbekistan** | 49840.07 (38781.57, 63867.16) | 326.56 (256.24, 412.78) | 102091.82 (78239.59, 132522.57) | 326.47 (256.03, 412.57) | -0.03 (-0.17, 0.08) |
| **Vanuatu** | 716.19 (557.28, 926.38) | 674.77 (533.98, 854.6) | 1620.44 (1272.88, 2088.8) | 676.28 (535.4, 855.86) | 0.22 (0.11, 0.35) |
| **Venezuela (Bolivarian Republic of)** | 45712.56 (35435.01, 59699.81) | 313.46 (244.33, 397.21) | 94918.17 (73593.97, 121158.37) | 313.57 (244.46, 397.34) | 0.04 (0, 0.08) |
| **Viet Nam** | 402183.77 (313676.25, 514128.99) | 778.97 (613.44, 992.84) | 842272.99 (652173.41, 1086995.37) | 776.93 (611.27, 990.33) | -0.26 (-0.43, -0.09) |
| **Yemen** | 48422.76 (37682.01, 62565.29) | 601.95 (473.41, 775.32) | 138483.17 (107024.16, 181749.34) | 602.56 (473.66, 774.31) | 0.1 (-0.28, 0.37) |
| **Zambia** | 13114.77 (10030.48, 16840.05) | 267.87 (208.32, 339.93) | 33726.53 (25605.53, 43989.35) | 267.66 (208.63, 339.72) | -0.08 (-0.31, 0.15) |
| **Zimbabwe** | 17670.31 (13614.16, 22620.64) | 268.22 (208.82, 340.4) | 30281.72 (23169.48, 39313.93) | 269.43 (209.91, 342.02) | 0.45 (0.21, 0.86) |
| **YLDs** | | | | | |
| **Afghanistan** | 22100.06 (14508.11, 32046.17) | 280.65 (187.04, 408.15) | 62138.18 (40157.8, 90270.5) | 277.73 (184.49, 403.59) | -1.04 (-3.69, 1.58) |
| **Albania** | 3790.53 (2472.86, 5525.06) | 140.28 (91.72, 201.86) | 4817.08 (3131.05, 6971.37) | 141.15 (92.54, 205.3) | 0.62 (-2.88, 4.22) |
| **Algeria** | 46454.95 (30525.76, 67284.9) | 285.53 (189.48, 412.69) | 117900.71 (76888.29, 172558.92) | 283.99 (186.9, 411.29) | -0.54 (-2.96, 1.86) |
| **American Samoa** | 109.54 (70.8, 156.85) | 317.01 (205.86, 454.37) | 165.82 (108.76, 239.95) | 316.6 (207.22, 455.44) | -0.13 (-2.36, 2.21) |
| **Andorra** | 214.83 (142.48, 308.23) | 352.18 (233.46, 509.56) | 398.83 (259.11, 578.39) | 353.53 (232.56, 512.12) | 0.38 (-1.82, 2.64) |
| **Angola** | 7253.4 (4747.25, 10577.69) | 112.93 (74.99, 161.99) | 21482.62 (13990.32, 31550.83) | 114.11 (75.63, 165) | 1.05 (-2.86, 4.92) |
| **Antigua and Barbuda** | 72.53 (47.65, 104.39) | 135.73 (89.1, 196.71) | 142.4 (92.72, 207.13) | 135.05 (88.46, 193.81) | -0.5 (-4.16, 2.97) |
| **Argentina** | 61785.78 (40473.38, 87880.33) | 192.43 (125.73, 274.56) | 95349.56 (62523.24, 135862.24) | 192.24 (126.84, 273.44) | -0.1 (-2.92, 2.94) |
| **Armenia** | 4401.42 (2883.1, 6468.62) | 141.06 (92.32, 204.37) | 5185.16 (3395.41, 7490.42) | 141.41 (92.4, 204.84) | 0.25 (-3.4, 4.05) |
| **Australia** | 19965.78 (13093.47, 28642.19) | 107.88 (70.51, 154.18) | 33602.12 (21711.5, 48524.79) | 107.86 (70.81, 155.14) | -0.02 (-4.03, 4.19) |
| **Austria** | 32663.53 (21346.07, 47378.18) | 356.43 (234.66, 513.92) | 41227.67 (26880.45, 59663.84) | 355.25 (234.14, 515.17) | -0.33 (-2.64, 2.11) |
| **Azerbaijan** | 8566.62 (5548.83, 12537.58) | 141.9 (92.2, 203.57) | 15951.44 (10299.59, 23437.9) | 141.31 (92.25, 204) | -0.42 (-3.94, 3.19) |
| **Bahamas** | 285.28 (184.78, 412.26) | 135.59 (89.74, 195.2) | 580.3 (375.44, 845.07) | 135.24 (88.87, 195.33) | -0.26 (-3.65, 3.53) |
| **Bahrain** | 1040.61 (669.66, 1568.8) | 270.13 (179.07, 391.87) | 4834.37 (3128.47, 7160.09) | 264.11 (175.41, 382.45) | -2.23 (-4.78, 0.51) |
| **Bangladesh** | 96135.95 (62515.27, 140657.22) | 138.47 (90.65, 201.73) | 212193.65 (138025.31, 305491.1) | 139.67 (91.19, 201.36) | 0.87 (-2.48, 4.4) |
| **Barbados** | 348.67 (230.72, 499.66) | 136.19 (90.38, 196.87) | 531.44 (346.61, 776.79) | 135.53 (88.99, 196.01) | -0.49 (-4.04, 2.82) |
| **Belarus** | 16659.76 (10920.09, 24104.44) | 140.71 (91.66, 202.11) | 17946.95 (11615.37, 25994.24) | 141.13 (91.2, 203.8) | 0.3 (-3.24, 4.01) |
| **Belgium** | 35176.03 (23634.41, 50793.88) | 297.05 (200.27, 432.19) | 42719.8 (28673.37, 62296.17) | 296.15 (198.44, 430.52) | -0.3 (-2.53, 2.25) |
| **Belize** | 161.05 (105.76, 234.95) | 135.29 (88.84, 195.17) | 488.69 (318.38, 701.98) | 134.77 (90.38, 194.99) | -0.38 (-3.63, 3.18) |
| **Benin** | 3304.02 (2155.68, 4789.91) | 117.72 (77.25, 169.33) | 9237.35 (6038.28, 13317.1) | 118.09 (78.25, 170.26) | 0.31 (-3.43, 4.44) |
| **Bermuda** | 91.66 (59.84, 133.63) | 136.14 (89.4, 196.54) | 126.15 (81.94, 181.95) | 135.8 (89.55, 196.35) | -0.25 (-3.78, 3.55) |
| **Bhutan** | 549.35 (356.79, 800.23) | 138.93 (90.04, 200.21) | 995.54 (642.36, 1460.49) | 139.25 (90.88, 201.91) | 0.23 (-3.4, 4.05) |
| **Bolivia (Plurinational State of)** | 5939.86 (3864.55, 8668.36) | 134.45 (89.29, 195.3) | 14228.76 (9487.61, 20517.7) | 134.77 (89.6, 194.02) | 0.24 (-3.2, 4.17) |
| **Bosnia and Herzegovina** | 6583.94 (4287.04, 9648.75) | 140.64 (91.4, 203.77) | 6384.61 (4143.45, 9225.37) | 140.12 (91.39, 202.19) | -0.37 (-4.07, 3.39) |
| **Botswana** | 949.51 (611.31, 1373.99) | 114.03 (74.62, 164.51) | 2338.63 (1540.02, 3419.8) | 112.67 (74.16, 161.44) | -1.2 (-4.87, 3.15) |
| **Brazil** | 261487.05 (170711.75, 381181.87) | 221.32 (145.14, 321.67) | 542596.52 (356801.63, 792398.19) | 221.72 (145.36, 322.57) | 0.18 (-0.88, 1.22) |
| **Brunei Darussalam** | 313.66 (202.12, 458.83) | 168.26 (109.91, 242.21) | 778.25 (506.29, 1138.15) | 170.23 (112.38, 244.65) | 1.17 (-1.8, 4.47) |
| **Bulgaria** | 15016.7 (9811.82, 21728.06) | 140.45 (92.13, 203.15) | 14031.26 (9086.12, 20004.78) | 140.63 (91.04, 202.2) | 0.13 (-3.42, 3.66) |
| **Burkina Faso** | 6831.23 (4498.12, 9846.36) | 117.7 (77.8, 168.72) | 16933.05 (11102.16, 24623.67) | 118.85 (77.55, 171.4) | 0.98 (-2.78, 5.18) |
| **Burundi** | 3857.86 (2497.21, 5578.22) | 113.91 (74.87, 164.49) | 8477.44 (5446.07, 12310.72) | 112.61 (73.96, 162.06) | -1.14 (-4.67, 2.83) |
| **Cabo Verde** | 276.7 (182.43, 396.35) | 120.76 (80.87, 174.27) | 620.06 (403.09, 897.46) | 118.32 (78.52, 171.42) | -2.02 (-6.2, 2.24) |
| **Cambodia** | 24227.77 (16029.44, 35256.28) | 379.07 (251.21, 554.09) | 56599.8 (37085.89, 82740.42) | 379.61 (251.28, 550.94) | 0.14 (-2.05, 2.52) |
| **Cameroon** | 7595.9 (4995.83, 10980.62) | 116.99 (77.4, 168.08) | 22904.96 (14743.3, 33436.37) | 117.4 (77.54, 169.15) | 0.35 (-3.49, 4.01) |
| **Canada** | 90595.7 (59616.87, 129851.64) | 292.87 (192.28, 423.36) | 144834.01 (95234.21, 210106.99) | 292.18 (191.39, 419.7) | -0.24 (-2.57, 2.05) |
| **Central African Republic** | 1992.29 (1284.54, 2907.8) | 112.71 (73.99, 162.08) | 4037.66 (2584.22, 5900.7) | 113.12 (74.14, 162.78) | 0.37 (-3.89, 4.67) |
| **Chad** | 4311.94 (2790.62, 6250.09) | 118.02 (77.9, 170.15) | 10561.69 (6813.76, 15297.87) | 116.82 (77.44, 168.68) | -1.02 (-4.99, 3.11) |
| **Chile** | 22759.12 (15005.42, 32579.77) | 192 (126.76, 274.71) | 41651.56 (27179.99, 60077.01) | 191.63 (126.24, 275.07) | -0.19 (-3.1, 3.01) |
| **China** | 3814002.2 (2479006.8, 5573875.98) | 352.84 (230.02, 516.83) | 6791996.75 (4412004.83, 9788048.76) | 358.13 (234.54, 515.95) | 1.5 (-4.27, 7.29) |
| **Colombia** | 33342.4 (21806.62, 48949.68) | 134.7 (87.75, 194.95) | 70214.23 (46582.44, 102460.81) | 135.53 (89.98, 196.88) | 0.62 (-3.06, 4.09) |
| **Comoros** | 335.31 (219.42, 484.5) | 113.82 (74.62, 163.93) | 702.29 (456.81, 1023.88) | 114.45 (74.63, 165.65) | 0.55 (-3.31, 4.7) |
| **Congo** | 1743.43 (1131.57, 2526.2) | 113.49 (74.46, 163.22) | 4662.07 (2986.62, 6849.18) | 113.27 (74.22, 162.01) | -0.19 (-3.73, 3.59) |
| **Cook Islands** | 49.03 (31.9, 70.24) | 318.21 (208.44, 459.97) | 67.22 (43.42, 96.32) | 318.94 (207.33, 456.2) | 0.23 (-2.01, 2.72) |
| **Costa Rica** | 3109.86 (2008.79, 4556.64) | 135.01 (88.58, 195.12) | 7030.35 (4643.91, 10163.74) | 135.25 (89.3, 195.73) | 0.18 (-3.39, 3.83) |
| **Croatia** | 8252.81 (5363.55, 11960.35) | 140.22 (91.68, 203.54) | 8352.01 (5443.9, 11996.81) | 140.21 (91.44, 202.65) | -0.01 (-3.63, 3.55) |
| **Cuba** | 14512.23 (9635.67, 20814.85) | 134.75 (89.63, 194.41) | 20639.54 (13460.99, 29955.54) | 134.91 (88.82, 195.81) | 0.12 (-3.65, 3.85) |
| **Cyprus** | 2887.45 (1886.94, 4187.45) | 356.08 (231.88, 515.55) | 5775.82 (3812.01, 8367.67) | 356.98 (233.82, 515.2) | 0.25 (-2.16, 2.61) |
| **Czechia** | 17036.21 (11231.81, 24441.05) | 140.2 (91.56, 202.26) | 20764.29 (13629.23, 29760.51) | 139.7 (91.55, 201.13) | -0.36 (-3.82, 3.17) |
| **Côte d'Ivoire** | 8352.09 (5430.7, 12188.35) | 115.14 (76.52, 166.25) | 21206.09 (13755.78, 30943.05) | 116.26 (76.9, 165.97) | 0.98 (-2.93, 4.94) |
| **Democratic People's Republic of Korea** | 62054.25 (40287.31, 91019.83) | 321.03 (208.44, 464.39) | 100950.67 (66725.99, 148360.71) | 317.22 (208.13, 462.08) | -1.19 (-3.6, 1.26) |
| **Democratic Republic of the Congo** | 26621.05 (17484.1, 38625.58) | 112.65 (74.45, 162.1) | 65178.74 (42514.22, 94761.7) | 113.24 (74.58, 163.74) | 0.52 (-3.28, 4.36) |
| **Denmark** | 27611.19 (18671.29, 39930.12) | 446.9 (299.25, 645.48) | 32653.82 (21563.91, 47048.08) | 446.46 (298.43, 647.4) | -0.1 (-2.1, 2.01) |
| **Djibouti** | 322.47 (209.68, 476.03) | 113.49 (75.62, 163.24) | 1126.86 (735.58, 1656.08) | 113.31 (74.08, 162.39) | -0.15 (-4.1, 3.75) |
| **Dominica** | 85.7 (56.59, 123.57) | 135.58 (88.97, 196.78) | 106.67 (70.53, 153.29) | 134.26 (88.54, 193.89) | -0.97 (-4.55, 2.76) |
| **Dominican Republic** | 6940.19 (4546.94, 10051.37) | 135.43 (89.55, 194.63) | 14169.27 (9370.17, 20368.07) | 135.11 (89.8, 194.34) | -0.23 (-3.76, 3.22) |
| **Ecuador** | 9754.21 (6307.14, 14240.18) | 135.1 (89, 194.51) | 22617.03 (14941.79, 32699.38) | 135.2 (89.11, 195.49) | 0.08 (-3.47, 3.65) |
| **Egypt** | 113338.84 (74249.36, 165950.72) | 284.16 (189.07, 415.58) | 239226.4 (156647.14, 353108.32) | 281.23 (186.53, 412.03) | -1.03 (-3.68, 1.45) |
| **El Salvador** | 4992.76 (3295.94, 7269.71) | 134.66 (89.14, 194.48) | 8148.07 (5373.19, 11846.34) | 135.16 (89.03, 196.53) | 0.38 (-3.49, 3.98) |
| **Equatorial Guinea** | 306.22 (202.34, 441.92) | 112.95 (74.65, 161.54) | 1026.65 (666.77, 1505.65) | 113.92 (75.54, 162.99) | 0.86 (-3.16, 5.04) |
| **Eritrea** | 2009.6 (1290.14, 2963.86) | 113.5 (75.4, 163.75) | 5290.02 (3423.73, 7779.1) | 113.67 (74.48, 164.21) | 0.16 (-3.74, 4.1) |
| **Estonia** | 2566.94 (1680.67, 3729.67) | 140.58 (92.08, 203.22) | 2528.49 (1650.58, 3635.75) | 141.03 (92.63, 204.95) | 0.32 (-3.04, 3.92) |
| **Eswatini** | 537.93 (349.19, 788.24) | 114.18 (75.48, 164.91) | 973.56 (638.01, 1414.69) | 112.51 (74.27, 161.63) | -1.46 (-5.4, 2.91) |
| **Ethiopia** | 41154.22 (26700.87, 60041.9) | 133.42 (87.82, 190.57) | 92490.78 (59626.06, 134191.75) | 134.51 (87.84, 191.31) | 0.82 (-0.99, 2.68) |
| **Fiji** | 1815.8 (1163.99, 2612.29) | 318.44 (207.85, 457.31) | 2823.18 (1856.93, 4071.86) | 316.45 (206.72, 452.75) | -0.62 (-2.87, 1.8) |
| **Finland** | 25632.4 (17189.38, 37144.11) | 429.8 (290.19, 628.45) | 30554.17 (20410.3, 43526.95) | 428.8 (288.49, 611.15) | -0.23 (-7.06, 6.39) |
| **France** | 183812.21 (123084.45, 263739.83) | 278.95 (187.04, 405.92) | 231701.18 (153012.07, 333582.36) | 279.23 (185.91, 406.67) | 0.1 (-2.39, 2.67) |
| **Gabon** | 782.14 (513.92, 1133.63) | 113.45 (75.34, 163.4) | 1651.94 (1080.62, 2416.68) | 113.44 (75.36, 164.06) | -0.01 (-3.93, 4.01) |
| **Gambia** | 674.35 (435.91, 974.65) | 116.32 (77.67, 166.74) | 1745.49 (1124.96, 2516.7) | 117.44 (77.16, 168.82) | 0.96 (-2.78, 4.93) |
| **Georgia** | 8405.22 (5471.36, 12116.94) | 141.99 (92.27, 205.01) | 6553.86 (4275.64, 9459.85) | 140.87 (92.11, 205.2) | -0.79 (-4.22, 2.81) |
| **Germany** | 348049.43 (228690.77, 507508.83) | 356.53 (233.19, 518.59) | 398497.26 (262693.56, 576722.17) | 353.8 (234.96, 510.13) | -0.77 (-3, 1.64) |
| **Ghana** | 11254.87 (7358.22, 16331.06) | 117.52 (77.27, 169.22) | 28741.5 (18586.95, 41560.26) | 118.85 (78.11, 171.24) | 1.13 (-2.82, 5.02) |
| **Greece** | 47431.65 (31392.33, 67919.57) | 388.44 (257.64, 560.69) | 53872.61 (35841.36, 77396.77) | 388.09 (259.18, 557.29) | -0.09 (-2.3, 2.1) |
| **Greenland** | 145.87 (95.71, 214.45) | 281.85 (185.46, 405.44) | 193.48 (127.56, 280.1) | 285.52 (187.69, 410.53) | 1.3 (-1.26, 4.11) |
| **Grenada** | 90.66 (59.66, 130.16) | 135.64 (89.39, 195.94) | 154.34 (101.18, 225.18) | 134.27 (89.77, 192.6) | -1.01 (-4.35, 2.66) |
| **Guam** | 370.04 (237.92, 532.3) | 319.78 (208.42, 459.63) | 580.29 (375.69, 836.56) | 319.8 (207.5, 459.68) | 0.01 (-2.22, 2.57) |
| **Guatemala** | 6867.71 (4407.25, 10052.83) | 133.75 (87.91, 193.31) | 19274.94 (12729.6, 28196.96) | 134.53 (88.69, 194.73) | 0.58 (-3.05, 4.39) |
| **Guinea** | 4863.89 (3213.42, 6991.25) | 117.68 (77.66, 168.8) | 9447.73 (6134.95, 13619.96) | 117.9 (77.59, 169.57) | 0.19 (-3.57, 4.17) |
| **Guinea-Bissau** | 712.46 (465.92, 1035.89) | 117.82 (77.82, 169.94) | 1462.08 (954.35, 2105.64) | 118.14 (77.94, 169.83) | 0.27 (-3.72, 4.2) |
| **Guyana** | 739.85 (479.85, 1082.72) | 133.34 (88.6, 192.56) | 987.6 (644.95, 1434.94) | 133.31 (88.78, 192.17) | -0.02 (-4.03, 3.71) |
| **Haiti** | 5947.29 (3911.53, 8625.33) | 134.03 (88.66, 194.33) | 13462.18 (8662.8, 19717.08) | 133.8 (87.31, 192.79) | -0.17 (-3.77, 3.49) |
| **Honduras** | 3901.9 (2534.51, 5705.2) | 134.66 (89.1, 195.17) | 10680.88 (6919.46, 15637.14) | 134.82 (88.3, 194.73) | 0.11 (-3.36, 3.77) |
| **Hungary** | 17708.38 (11508.24, 25583.96) | 140.2 (91.46, 202.65) | 19194.81 (12615.63, 27477.48) | 140.52 (91.65, 203.05) | 0.23 (-3.31, 4.11) |
| **Iceland** | 932.57 (617.37, 1353.33) | 355.93 (233.67, 517.89) | 1452.32 (949.2, 2102.69) | 354.95 (232.77, 512.21) | -0.27 (-2.62, 2.04) |
| **India** | 1044693.51 (685430.85, 1528223.14) | 160.57 (107.1, 230.62) | 2176947.65 (1449331.77, 3137620.89) | 161.95 (107.99, 234.14) | 0.86 (0.15, 1.58) |
| **Indonesia** | 599748.45 (389890.56, 863211.6) | 426.01 (279.88, 613.84) | 1161438.76 (757377.86, 1691309.9) | 427.53 (280.32, 613.71) | 0.36 (-0.4, 1.08) |
| **Iran (Islamic Republic of)** | 154692.14 (100655.62, 224784.05) | 418.85 (277.1, 604.78) | 385175.02 (253142.92, 558606.79) | 423.54 (280.26, 609.82) | 1.12 (0.44, 1.79) |
| **Iraq** | 29896.38 (19523.38, 43115.93) | 280.68 (186.85, 406.22) | 94611.24 (62325.65, 137968.22) | 281.54 (186.75, 409.52) | 0.31 (-2.13, 2.81) |
| **Ireland** | 13065.96 (8603.1, 18944.49) | 355.55 (233.31, 518.19) | 20865.84 (13780.38, 30128.18) | 355.47 (236.57, 516.66) | -0.02 (-2.26, 2.43) |
| **Israel** | 16914.85 (11213.75, 24408.06) | 358.03 (236.77, 518.21) | 34502.79 (22582.07, 49838.32) | 356.93 (232.36, 514.32) | -0.3 (-2.52, 1.97) |
| **Italy** | 262738.41 (173653.59, 374755.45) | 383.7 (253.57, 545.79) | 313203.51 (208928.87, 447449.06) | 384.17 (254.24, 546.39) | 0.12 (-0.6, 0.85) |
| **Jamaica** | 2511.4 (1637.84, 3622.16) | 135.7 (88.5, 196.41) | 4051.67 (2663.99, 5815.29) | 134.86 (89.38, 194.52) | -0.62 (-4.14, 2.8) |
| **Japan** | 307770.45 (199809.25, 449601.82) | 195.82 (129.34, 286.66) | 377560.06 (248509.82, 546899.63) | 195.78 (129.81, 287.92) | -0.02 (-0.78, 0.65) |
| **Jordan** | 6107.16 (3984.5, 8882.7) | 281.73 (186.55, 407.26) | 27464 (17726.83, 40291.69) | 279.58 (185.86, 407.1) | -0.76 (-3.3, 1.66) |
| **Kazakhstan** | 20683.9 (13500.87, 30036.9) | 141.11 (91.75, 203.09) | 26937.11 (17616.71, 39617.89) | 140.81 (92.04, 203.53) | -0.21 (-3.6, 3.44) |
| **Kenya** | 17618.74 (11417.28, 25623.19) | 134.16 (88.08, 191.76) | 48448.12 (31405.28, 70243.2) | 134.5 (88.58, 191.49) | 0.26 (-0.64, 1.06) |
| **Kiribati** | 168.6 (109.27, 241.44) | 318.54 (208.65, 453.89) | 307.99 (200.2, 444.18) | 319.6 (209.36, 461.37) | 0.33 (-1.89, 2.81) |
| **Kuwait** | 2865.21 (1887.64, 4349.87) | 209.14 (137.67, 306.43) | 10849.71 (6978.32, 16598.96) | 215.03 (141.02, 314.12) | 2.82 (-0.21, 6.06) |
| **Kyrgyzstan** | 4903.8 (3221.11, 7118) | 141.3 (92.62, 203.68) | 8266.76 (5303.81, 12100.97) | 141.69 (91.85, 204.35) | 0.28 (-3.31, 3.9) |
| **Lao People's Democratic Republic** | 10435.78 (6941.84, 15124.96) | 378.15 (252.47, 549.19) | 22999.4 (15116.21, 33668.87) | 378.98 (250.78, 550.69) | 0.22 (-2.02, 2.46) |
| **Latvia** | 4407.49 (2875.87, 6364.52) | 140.33 (92.08, 202.76) | 3760.7 (2424.58, 5422.47) | 140.99 (91.83, 204.88) | 0.47 (-3.21, 4.04) |
| **Lebanon** | 7477.56 (4924.42, 10874.21) | 285.1 (188.17, 412.62) | 15453.04 (10265.57, 22581.39) | 287.84 (188.98, 422.2) | 0.96 (-1.63, 3.47) |
| **Lesotho** | 1431.4 (932.13, 2053.6) | 113.97 (74.09, 163.6) | 1960.14 (1296.11, 2831.92) | 112.32 (74.72, 160.38) | -1.44 (-5.33, 2.62) |
| **Liberia** | 1546.28 (1026.17, 2225.84) | 114.94 (75.24, 164.88) | 3995.41 (2614.06, 5847.39) | 115.42 (76.2, 165.5) | 0.42 (-3.31, 4.4) |
| **Libya** | 7236.58 (4755.43, 10500.24) | 277.41 (183.84, 402.31) | 20061.22 (13012.93, 29522.87) | 280.73 (187.68, 407.48) | 1.2 (-1.32, 3.77) |
| **Lithuania** | 5796.54 (3770.24, 8340.09) | 140.15 (92.32, 202.78) | 5466.25 (3504.02, 7859.61) | 140.9 (91.34, 204.53) | 0.53 (-3.01, 3.85) |
| **Luxembourg** | 1618.95 (1067.71, 2336.73) | 355.73 (235, 512.61) | 2755.35 (1799.91, 3999.89) | 353.79 (234.16, 515.18) | -0.54 (-2.83, 1.42) |
| **Madagascar** | 8394.04 (5448.82, 12145.2) | 113.27 (73.76, 162.67) | 20901.05 (13556.37, 30497.59) | 114.05 (75.33, 163.23) | 0.7 (-3.09, 4.76) |
| **Malawi** | 6611.3 (4308.62, 9657.24) | 113.24 (74.09, 164.75) | 13412.86 (8709.14, 19537.47) | 113.82 (74.47, 163.27) | 0.52 (-3.25, 4.59) |
| **Malaysia** | 34994.61 (22881.9, 49854.34) | 267.19 (176.46, 376.67) | 94628.08 (62812.15, 138635.3) | 299.87 (200.25, 437.37) | 12.23 (2.99, 23.31) |
| **Maldives** | 495.33 (325.04, 729.39) | 376.34 (248.07, 548.87) | 1898.2 (1229.68, 2809.25) | 376.19 (247.72, 547.76) | -0.04 (-2.18, 2.23) |
| **Mali** | 6489.86 (4286.93, 9441.25) | 117.06 (77.01, 170.26) | 15342.59 (9981.25, 22087.18) | 117.52 (77.33, 168.32) | 0.39 (-3.55, 4.96) |
| **Malta** | 1463.32 (961.53, 2114.37) | 357.27 (234.49, 516.79) | 2037.16 (1360.45, 2950.58) | 354.71 (235.43, 514.84) | -0.72 (-2.86, 1.55) |
| **Marshall Islands** | 83.16 (54.17, 119.82) | 317.49 (206.78, 457.43) | 156.72 (101.08, 227.13) | 315.76 (207.02, 452.23) | -0.54 (-2.91, 1.73) |
| **Mauritania** | 1576.33 (1029.2, 2275.98) | 118.32 (77.98, 170.23) | 3381.01 (2214.05, 4898.82) | 118.54 (77.41, 171.44) | 0.18 (-3.47, 4.22) |
| **Mauritius** | 3603.72 (2370.46, 5257.54) | 378.05 (251.29, 552.15) | 6150.9 (4057.76, 8962.71) | 376.28 (250.91, 547.85) | -0.47 (-2.5, 1.68) |
| **Mexico** | 94960.78 (62331.15, 138532.78) | 157.47 (104.76, 227.83) | 202653.99 (134734.7, 296442.6) | 157.83 (104.51, 229.16) | 0.23 (-0.63, 1.09) |
| **Micronesia (Federated States of)** | 210.63 (137.22, 300.17) | 318.24 (207, 455.68) | 289.35 (187.67, 417.53) | 318.36 (208.32, 456.26) | 0.04 (-2.35, 2.29) |
| **Monaco** | 148.42 (97.68, 211.44) | 358.01 (235.22, 520.17) | 184.2 (121.06, 265.9) | 356.32 (232.02, 513.74) | -0.47 (-2.63, 1.78) |
| **Mongolia** | 2017.79 (1299.59, 2945.87) | 140.84 (91.21, 203.56) | 4628.69 (2974.27, 6795.17) | 141.28 (91.57, 203.84) | 0.31 (-3.06, 4.1) |
| **Montenegro** | 909.67 (588.08, 1330.32) | 140.92 (91.14, 205.06) | 1122.74 (730.63, 1631.42) | 140.56 (92.13, 205.62) | -0.26 (-3.79, 3.28) |
| **Morocco** | 51522.47 (33656.08, 74908.05) | 285.33 (187.57, 416.59) | 104105.83 (68398.72, 152467.11) | 283.97 (188.13, 414.42) | -0.48 (-2.82, 1.93) |
| **Mozambique** | 9648.72 (6411.15, 13898.69) | 113.03 (75.76, 162.23) | 20142.11 (13133.42, 29431.7) | 113.31 (74.44, 163.13) | 0.25 (-3.77, 4.25) |
| **Myanmar** | 114233.91 (74762.54, 164507.56) | 377.74 (247.91, 548.51) | 206279.76 (135511.48, 301417.54) | 380.07 (252.17, 552.64) | 0.62 (-1.54, 2.87) |
| **Namibia** | 1071.82 (695.53, 1544.9) | 113.57 (74.56, 162.17) | 2182.06 (1432.02, 3143.12) | 113.51 (75.64, 161.05) | -0.05 (-3.67, 3.74) |
| **Nauru** | 21.65 (13.82, 31.36) | 318.16 (207.71, 456.92) | 24.97 (15.96, 36.27) | 320.14 (208.71, 461.2) | 0.62 (-1.85, 3.1) |
| **Nepal** | 18478.87 (12051.44, 27217.79) | 138.06 (91.02, 200.53) | 37424.03 (24095.35, 54104.06) | 139.65 (89.36, 200.61) | 1.15 (-2.4, 5.02) |
| **Netherlands** | 55640.5 (36812.84, 80597.01) | 323.4 (213.33, 470.38) | 70716.58 (46204.96, 102099.27) | 322.49 (212.27, 467.28) | -0.28 (-2.48, 1.94) |
| **New Zealand** | 4200.19 (2729.68, 6160.04) | 114.65 (74.82, 169.1) | 4897.83 (3259.16, 6911.84) | 86.06 (56.47, 122.89) | -24.94 (-32.35, -16.76) |
| **Nicaragua** | 3088.9 (2007.72, 4529.55) | 134.58 (88.59, 194.09) | 7756.99 (5078.33, 11298.93) | 134.93 (89.12, 194.4) | 0.25 (-3.41, 4.16) |
| **Niger** | 5332.44 (3473.81, 7728.53) | 117.01 (77.19, 168.37) | 14730.08 (9592.55, 21234.29) | 118.48 (78.77, 169.63) | 1.25 (-2.63, 5.16) |
| **Nigeria** | 84565.61 (54833.82, 122277.51) | 139.84 (92.49, 201.98) | 205478.4 (133133.16, 299690.34) | 147.77 (96.27, 213.1) | 5.68 (1.09, 10.57) |
| **Niue** | 6.56 (4.27, 9.41) | 320.5 (207.78, 460.51) | 6.08 (3.92, 8.78) | 317.46 (206.72, 456.22) | -0.95 (-3.09, 1.65) |
| **North Macedonia** | 2871.55 (1872.95, 4178.63) | 140.47 (91.35, 203.95) | 3943.53 (2576.14, 5724.62) | 140.24 (91.27, 202.71) | -0.17 (-3.62, 3.41) |
| **Northern Mariana Islands** | 123.13 (78.81, 181.93) | 314.24 (203, 448.11) | 170.76 (108.81, 253.42) | 318.17 (206.74, 456.86) | 1.25 (-1.16, 3.83) |
| **Norway** | 16042.8 (10637.99, 22956.52) | 321.32 (212.51, 465.85) | 17333.76 (11427.04, 25110.68) | 265.2 (174.84, 380.08) | -17.47 (-23.22, -10.99) |
| **Oman** | 3366.48 (2170.9, 4991.64) | 268.89 (179.12, 389.57) | 11150.07 (7101.51, 16784.14) | 265.01 (178.77, 383.8) | -1.44 (-4.1, 1.1) |
| **Pakistan** | 121344.97 (80397.11, 176673.52) | 162.68 (107.97, 235.81) | 267531.48 (174668.08, 393466.33) | 162.87 (107.95, 235.97) | 0.11 (-1.84, 2.13) |
| **Palau** | 41.77 (27.11, 59.84) | 319.01 (207.46, 457.86) | 72.56 (46.62, 105.73) | 314.75 (205.18, 449.05) | -1.34 (-3.61, 1.13) |
| **Palestine** | 3299.88 (2160.43, 4776.52) | 287.75 (189.99, 414.7) | 10108.69 (6549.07, 14844.3) | 281.72 (185.6, 412.07) | -2.09 (-4.73, 0.67) |
| **Panama** | 2545.31 (1654.05, 3692.81) | 134.82 (88.48, 194.3) | 5671.7 (3741.38, 8190.27) | 134.74 (89.18, 193.91) | -0.06 (-3.79, 3.74) |
| **Papua New Guinea** | 8847.29 (5675.87, 12681.34) | 315.51 (205.61, 455.63) | 23706.09 (15262, 33886.85) | 315.4 (206.46, 449.11) | -0.03 (-2.39, 2.36) |
| **Paraguay** | 4681.46 (3046.58, 6819) | 164.61 (108.32, 239.69) | 10622.92 (6906.24, 15375.51) | 164.29 (107.15, 237.97) | -0.2 (-3.32, 3.32) |
| **Peru** | 21405.43 (13740.06, 31196.63) | 135.19 (88.01, 195.48) | 46419.84 (30404.02, 66807.32) | 135.57 (89.14, 195.15) | 0.28 (-3.2, 4.09) |
| **Philippines** | 231505.75 (152967.86, 338417.24) | 528.13 (349.57, 760.16) | 525552.08 (345862.98, 766353.09) | 530.07 (350.59, 764.8) | 0.37 (-0.13, 0.82) |
| **Poland** | 68643.05 (45571.76, 99280.61) | 164.06 (108.86, 236.34) | 86640.27 (56630.4, 125217.47) | 165.05 (109.52, 239.48) | 0.6 (-0.4, 1.59) |
| **Portugal** | 41138.33 (27400.73, 59681.07) | 356.53 (233.38, 516.95) | 51440.39 (33715.32, 74087.71) | 357.15 (236.75, 519.85) | 0.17 (-2.01, 2.26) |
| **Puerto Rico** | 4875.42 (3209.44, 6993.02) | 135.29 (89.06, 194.93) | 6439.4 (4302.15, 9257.01) | 135.16 (88.58, 193.74) | -0.09 (-3.66, 3.85) |
| **Qatar** | 953.92 (609.61, 1464.54) | 255.57 (169.32, 372.65) | 7559.6 (4851.63, 11433.14) | 247.62 (165.92, 357.44) | -3.11 (-5.9, -0.38) |
| **Republic of Korea** | 69942.12 (46032.26, 100424.73) | 172.29 (113.62, 247.81) | 132335.85 (86384.5, 194651.27) | 171.97 (112.56, 247.48) | -0.19 (-3.33, 3.02) |
| **Republic of Moldova** | 6384.6 (4207, 9230.78) | 140.38 (92.16, 201.99) | 6900.12 (4525.62, 10003.79) | 140.82 (92.27, 203.64) | 0.31 (-3.16, 4) |
| **Romania** | 36528.62 (23942.37, 53169.43) | 140.2 (92.15, 203.42) | 37643.02 (24558.04, 54615.8) | 140.84 (91.21, 205.25) | 0.46 (-3.05, 4.09) |
| **Russian Federation** | 280115.76 (184116.25, 408459.46) | 164.23 (108.73, 237.34) | 316503.24 (206819.75, 456661.41) | 164.9 (109.57, 238.37) | 0.41 (-0.45, 1.26) |
| **Rwanda** | 4900.03 (3169.61, 7078.85) | 113.83 (75.1, 163.95) | 10567.07 (6830.29, 15320.16) | 114.37 (74.59, 164.91) | 0.47 (-3.48, 4.78) |
| **Saint Kitts and Nevis** | 45.79 (30.03, 65.58) | 135.04 (87.99, 195.71) | 95.89 (61.61, 139.18) | 134.53 (88.78, 192.81) | -0.38 (-3.66, 3.36) |
| **Saint Lucia** | 135.44 (88.8, 196.22) | 134.76 (88.7, 193.54) | 284.04 (186.97, 414.28) | 134.24 (89.1, 194.03) | -0.38 (-3.91, 3.24) |
| **Saint Vincent and the Grenadines** | 108.06 (70.28, 155.77) | 135.11 (88.32, 196.43) | 173.69 (113.63, 253.18) | 134.05 (88.26, 194.17) | -0.78 (-4.46, 3.05) |
| **Samoa** | 357.57 (232.43, 517.31) | 319.74 (207.82, 458.05) | 556.01 (359.35, 805.09) | 318.07 (205.87, 455.16) | -0.52 (-2.93, 1.88) |
| **San Marino** | 96.1 (63.22, 139.18) | 356.28 (234.42, 518.38) | 150.73 (98.17, 218.38) | 358.1 (235.99, 517.64) | 0.51 (-1.82, 2.75) |
| **Sao Tome and Principe** | 90.29 (59.23, 130.57) | 118.43 (77.41, 170.17) | 187.01 (121.8, 272.12) | 117.92 (77.45, 169.46) | -0.43 (-4.26, 3.54) |
| **Saudi Arabia** | 27766.28 (18276.84, 40817.1) | 269.06 (178.15, 389.13) | 100131.77 (64563.4, 148661.79) | 268.59 (178.87, 389.36) | -0.18 (-2.7, 2.42) |
| **Senegal** | 5382.34 (3523.18, 7798.71) | 117.48 (77.91, 170.02) | 12519.7 (8185.79, 18112.85) | 117.96 (78.94, 169.26) | 0.41 (-3.64, 4.3) |
| **Serbia** | 15261.55 (9867.88, 22032.87) | 140.48 (91.69, 202.23) | 16299.51 (10744.07, 23335.89) | 140.3 (92.59, 203.46) | -0.13 (-3.67, 3.46) |
| **Seychelles** | 229.02 (150.02, 329.33) | 380.18 (250.33, 553.15) | 453.67 (298.6, 660.43) | 376.88 (250.45, 544.52) | -0.87 (-3.03, 1.5) |
| **Sierra Leone** | 2900.26 (1921.99, 4165.25) | 116.9 (77.49, 167.57) | 6582.07 (4275.42, 9572.11) | 117.23 (77.36, 168.5) | 0.29 (-3.66, 4.35) |
| **Singapore** | 5226.9 (3402.97, 7612.79) | 172.88 (112.76, 247.82) | 13399.14 (8738.75, 19502.61) | 172.18 (113.96, 248.75) | -0.41 (-3.58, 3) |
| **Slovakia** | 7980.47 (5203.55, 11524.67) | 140.33 (90.95, 202.64) | 10372.26 (6710.4, 14891.21) | 140.44 (91.48, 202.96) | 0.08 (-3.32, 3.67) |
| **Slovenia** | 3204.84 (2093.1, 4663.09) | 140.15 (91.76, 202.76) | 4119.77 (2643.94, 5922.02) | 140.06 (90.44, 202.11) | -0.06 (-3.54, 3.59) |
| **Solomon Islands** | 653.29 (419.44, 939.04) | 317.18 (205.48, 455.77) | 1529.5 (980.05, 2191.85) | 317.64 (208.38, 455.8) | 0.15 (-2.22, 2.51) |
| **Somalia** | 4974.35 (3246.39, 7315.96) | 113.4 (74.53, 162.81) | 13665.18 (8740.55, 20008.56) | 113.93 (74.6, 164.37) | 0.47 (-3.5, 4.63) |
| **South Africa** | 37803.34 (24556.04, 55221.08) | 134.5 (87.92, 190.86) | 72624.97 (47661.17, 105758.94) | 133.57 (88.16, 190.57) | -0.7 (-2.29, 0.84) |
| **South Sudan** | 4026.71 (2613.72, 5889.61) | 110.97 (73.41, 159.46) | 6814 (4419, 9951.03) | 112.12 (73.89, 160.59) | 1.04 (-3.08, 5.17) |
| **Spain** | 119202.05 (79657.27, 171579.17) | 270.48 (182.89, 386.79) | 165555.73 (110588.73, 240437.99) | 269.9 (181.06, 392.62) | -0.22 (-2.69, 2.41) |
| **Sri Lanka** | 54544.77 (35765.86, 79148.95) | 378.06 (250.03, 550.11) | 94561.13 (62630.52, 138196.71) | 378.74 (250.73, 554.59) | 0.18 (-2, 2.48) |
| **Sudan** | 35734.22 (23539.42, 51940) | 283.27 (187.64, 412.11) | 80487.43 (52630.59, 117694.42) | 282.31 (186.05, 410.82) | -0.34 (-2.92, 2.32) |
| **Suriname** | 423.41 (278.35, 615.39) | 134.78 (89.28, 194.65) | 831.8 (542.68, 1213.2) | 134.16 (88.44, 194.8) | -0.46 (-3.8, 3.19) |
| **Sweden** | 45080.74 (30155.39, 64680.47) | 433.01 (291.64, 624.78) | 55261.72 (37069.01, 79084.89) | 434.57 (291.85, 628.07) | 0.36 (-1.51, 2.14) |
| **Switzerland** | 29137.14 (19120.25, 42106.39) | 353.95 (231.32, 510.83) | 40377.21 (26298.55, 58448.28) | 354.71 (231.07, 511.38) | 0.21 (-2.07, 2.45) |
| **Syrian Arab Republic** | 20928.91 (13742.22, 30412.71) | 282.84 (186.38, 411.09) | 41033.61 (26713.67, 60136.87) | 285.23 (189.55, 415.68) | 0.84 (-1.69, 3.34) |
| **Taiwan (Province of China)** | 61557.31 (40363.51, 89478.8) | 317.77 (208.63, 464.73) | 98925.54 (66453.24, 137219.78) | 292.97 (197.84, 405.61) | -7.8 (-20.5, 7.32) |
| **Tajikistan** | 5015.99 (3245.05, 7360.64) | 141.37 (90.92, 206.04) | 10924.48 (6982.9, 16063.29) | 140.96 (90.7, 203.77) | -0.29 (-3.84, 3.26) |
| **Thailand** | 184097.79 (121366.77, 269566.71) | 378.76 (251.32, 551.82) | 361227.47 (237685.99, 530355.43) | 381.16 (253.4, 556.32) | 0.63 (-1.49, 2.88) |
| **Timor-Leste** | 1897.84 (1250.12, 2789.78) | 375.91 (249.12, 545.29) | 3691.31 (2422.03, 5331.99) | 376.45 (248.23, 549.81) | 0.14 (-1.91, 2.3) |
| **Togo** | 2462.17 (1603.45, 3565.31) | 118.32 (78.13, 170.44) | 6823.69 (4494.14, 9847.84) | 118.59 (78.15, 170.64) | 0.23 (-3.82, 4.31) |
| **Tokelau** | 4.19 (2.7, 5.93) | 323.47 (209.49, 461.09) | 4.24 (2.76, 6.07) | 318.39 (208.66, 456.13) | -1.57 (-3.85, 0.8) |
| **Tonga** | 218.12 (141.64, 312.87) | 321.36 (210.43, 459.98) | 278.06 (180.55, 398.31) | 320.57 (208.06, 459.08) | -0.25 (-2.49, 2.09) |
| **Trinidad and Tobago** | 1362.81 (898.42, 1953.99) | 134.72 (89.71, 192.6) | 2288.86 (1499.48, 3331.52) | 134.17 (87.92, 194.77) | -0.4 (-3.88, 3.19) |
| **Tunisia** | 17788.37 (11761.17, 25825.6) | 285.19 (188.1, 414.16) | 37855.99 (24817.5, 55427.32) | 285.97 (187.96, 415.09) | 0.27 (-2.06, 3.07) |
| **Turkey** | 130384.45 (85222.46, 191700.08) | 284.85 (187.91, 413.8) | 267435.12 (174136.91, 390415.91) | 284.83 (188.62, 414.82) | -0.01 (-2.43, 2.48) |
| **Turkmenistan** | 3628.18 (2347.13, 5326.16) | 141.65 (92.54, 206.4) | 6834.93 (4445.44, 10020.08) | 141.31 (91.85, 204.31) | -0.24 (-3.79, 3.32) |
| **Tuvalu** | 25.83 (16.81, 36.96) | 323.9 (209.95, 464.36) | 35.55 (23.11, 50.94) | 318.35 (206.75, 458.55) | -1.71 (-4.12, 0.64) |
| **Uganda** | 11041.14 (7230.42, 16079.57) | 112.48 (74.37, 161.17) | 27428.72 (17736.44, 40417.57) | 114.11 (74.18, 164.57) | 1.44 (-2.65, 5.49) |
| **Ukraine** | 102631.92 (67873.94, 148768.51) | 164.68 (110.4, 237.94) | 99265.46 (64969.74, 143905.51) | 165.35 (109.97, 237.9) | 0.4 (-2.74, 3.5) |
| **United Arab Emirates** | 3734.46 (2382.99, 5698.46) | 256.71 (171.45, 373.63) | 30439.68 (19270.28, 47075.6) | 250.68 (166.55, 364.05) | -2.35 (-5.31, 0.39) |
| **United Kingdom** | 307106.22 (206191.78, 439209.53) | 443.77 (298.35, 641.82) | 370075.82 (247710.52, 528171.87) | 446.79 (301.97, 636.94) | 0.68 (-4.84, 6.7) |
| **United Republic of Tanzania** | 17731.74 (11496.08, 26037.27) | 113.08 (74.28, 162.66) | 42894.05 (27838.35, 62036.23) | 114 (75.42, 163.63) | 0.82 (-2.95, 4.81) |
| **United States of America** | 1201621.37 (792529.02, 1709085.79) | 422.49 (279.7, 604.57) | 2043518.34 (1392664.75, 2886397.13) | 500.26 (338.92, 704.86) | 18.41 (9.89, 27.58) |
| **United States Virgin Islands** | 141.19 (91.21, 206.16) | 135.88 (88.52, 196.49) | 185.17 (120.75, 265.77) | 135.37 (88.01, 194.81) | -0.37 (-3.95, 3.39) |
| **Uruguay** | 6502.68 (4246.23, 9334.96) | 192.64 (126.88, 275.89) | 7976.21 (5224.38, 11376.75) | 192.58 (126.14, 276.69) | -0.03 (-2.88, 2.86) |
| **Uzbekistan** | 20526.22 (13307.23, 29994.51) | 141.35 (92.2, 205.08) | 43027.11 (27794.33, 63007.31) | 141.22 (92.24, 203.15) | -0.1 (-3.58, 3.47) |
| **Vanuatu** | 311.07 (199.61, 444.54) | 316.56 (206.87, 452.76) | 722.01 (470.74, 1031.92) | 317.15 (206.85, 456.96) | 0.18 (-2.15, 2.56) |
| **Venezuela (Bolivarian Republic of)** | 18644.91 (12039.07, 27026.73) | 134.84 (89.03, 194.63) | 41118.37 (26872.08, 59786.52) | 135.09 (88.89, 195.51) | 0.19 (-3.23, 3.79) |
| **Viet Nam** | 185286.05 (122428.53, 268597.81) | 382.44 (252.78, 557.27) | 412121.99 (269300.55, 604239.35) | 381.91 (251.97, 557.69) | -0.14 (-2.12, 1.79) |
| **Yemen** | 20692.02 (13572.78, 30310.61) | 282.12 (187.15, 407.25) | 59349.95 (38887.7, 86823.84) | 282.59 (186.49, 408.26) | 0.16 (-2.3, 2.49) |
| **Zambia** | 5167.83 (3313.2, 7553.42) | 113.15 (74.25, 162.46) | 13300.54 (8509.98, 19518.7) | 113.05 (74.39, 163.22) | -0.08 (-3.9, 4.1) |
| **Zimbabwe** | 7010.17 (4535.1, 10202.86) | 113.67 (73.69, 163.64) | 12128.49 (7867.29, 17638.76) | 113.71 (74.99, 162.88) | 0.04 (-3.79, 4.17) |

Legend. Data in parentheses are 95% uncertainty intervals (UI). YLD=years lived with a disability.
